# Phytogalactolipids activate humoral immunity against colorectal cancer

**DOI:** 10.1186/s13046-023-02660-x

**Published:** 2023-04-21

**Authors:** Han-Huei Lin, Yi-Shin Wu, Ting-Yan Jian, Jia-Yun Liao, Meng-Ting Chang, Lie-Fen Shyur, Yu-Ling Lin

**Affiliations:** 1 Agricultural Biotechnology Research Center, Academia Sinica, No. 128, Sec. 2, Academia Road, Nankang, Taipei 11529 Taiwan; 2 Program in Translational Medicine, College of Medicine, Kaohsiung Medical University, Kaohsiung, 807 Taiwan; 3 Institute of BioPharmaceutical Sciences, National Sun Yat-Sen University, Kaohsiung, 804 Taiwan; 4 Neuroscience Research Center, Taipei Medical University Hospital, Taipei, 110 Taiwan

**Keywords:** *Crassocephalum rabens*, Phytogalactolipid, Humoral immunity, IL-21, B cell differentiation

## Abstract

**Background:**

Colorectal cancer (CRC) is the third most lethal cancer in the world, and its incidence is steadily rising. In this study, we investigated the induction of humoral immunity by a phytogalactolipid enriched fraction (CRA) derived from the medicinal plant *Crassocephalum rabens (Benth.)* S. Moore to combat CRC.

**Methods:**

Immunocompetent BALB/c mice were used to evaluate CRA's therapeutic effects in CRC. The phenotypes of B cell subsets in splenocytes and tumors from the CRA-treated mice were isolated and analyzed by flow cytometry. The titers, isotypes, specificity, antigen recognition, and cytotoxic activity of CRA-induced anti-tumor antibodies were determined. The mechanisms of CRA on B cell differentiation were determined by cell-based analyses, including co-cultural with T cells, cytokine analysis, gene expression by qPCR, and protein expression by western blotting.

**Results:**

CRA efficiently inhibited tumor growth in colorectal tumor-bearing allograft mice. CRA treatment attracted an abundance of B cells into the tumor consequently enhancing the anti-tumor antibodies in sera and inducing a class-switch. CRA-induced antisera (designated CRA antisera) specifically recognized surface antigens on the plasma membrane of cancer cells. CRA antisera induced cytotoxicity including antibody-dependent cell cytotoxicity, phagocytosis, and complement-dependent cytotoxicity. CRA interacted with IL-6 receptor to activate STAT3 and cMaf, resulting in T cell secretion of IL-21, which, in turn induced B cell differentiation through the IL-21R/STAT3/Blimp-1 pathway.

**Conclusions:**

CRA regulated T cell activity resulting in B cell activation and triggering of anti-tumor antibodies to impede CRC progression.

**Supplementary Information:**

The online version contains supplementary material available at 10.1186/s13046-023-02660-x.

## Background

An increasing amount of research shows a strong positive correlation between the presence of B cells in tumors and cancer-specific survival and response to therapy [1, 2]. Anti-tumor auto-antibody (AA)-secreting tumor-infiltrating B cells (TIL-B) have been reported to improve clinical outcomes [2, 3]. A higher proportion of follicular B cells in tertiary lymphoid structures and tumor-infiltrating plasma cells is correlated with better long-term survival in cancer patients [2]. AA development has a protective role in antitumor immunity in cancer patients under treatment. Several AAs isolated from the plasma of cancer patients show anti-proliferative action. Selected tumor-specific AAs, such as SC-1 (stomach adenocarcinoma), CM-1/2 (colon adenocarcinoma), and PM-1/2 (pancreatic cancer) activated a caspase cascade and induced apoptotic cell death [4, 5]. Anti-TrkB autoantibody (clone no. 641) isolated from breast cancer patients also had an inhibitory effect on tumor growth in the MDA-MB-213 tumor-bearing mouse model [6]. IgG typed anti-GRP78 autoantibody isolated from the blood of cancer patients modulated cell proliferation and decreased invasiveness in prostate and ovarian cancer cells [7]. These anti-GRP78 autoantibodies were also able to block GRP78 signaling and enhance tissue factor procoagulant activity, thereby reducing the risk of cancer-related venous thromboembolism [7, 8]. Circulating anti-GRP78 autoantibodies which can recognize tumor antigens are used to coat nanoparticles to deliver drugs for ovarian cancer therapy [9]. Therefore, selected AAs have a high affinity to and recognize tumor antigens and are thus able to inhibit tumor growth.

*Crassocephalum rabens* (Benth.) S. Moore from the family Asteraceae also known as *Crassocephalum crepidioides* (Benth.) S. Moore, is a common vegetable and a popular folk medicine whose ethanolic extract has been shown to be a safe supplement [10]. *C. rabens* has attracted particular attention due to its prominent anti-inflammatory and anticancer activity [11–13]. The glyceroglycolipid, 1,2-di-*O*-α-linolenoyl-3-*O*-β-galactopyranosyl-*sn*-glycerol (dLGG) is a major component of *C. rabens* and has shown chemopreventive activity in cancer or sepsis by inhibiting inflammatory mediators, such as TNF-α, IL-6, or bioactive lipid mediator oxylipins [11, 12]. dLGG represses the metastatic ability of melanoma cells by deregulating epithelial-mesenchymal transition (EMT), and attenuating the tight junction permeability of pulmonary vasculature and circulating oxylipin dynamics [13]. dLGG used alone or in combination with doxorubicin effectively attenuates triple-negative breast cancer (TNBC) recurrence and lung metastasis through inhibiting the fatty acid binding protein (FABP)/epoxy-eicosatrienoic acid (EET)-mediated signaling axes [12]. However, the immunomodulation of phytogalactolipid from *C. rabens* by triggering the humoral immune response against colorectal cancer has never been reported.

In this study, the bioefficacy of CRA was demonstrated not to be limited to cytotoxicity to CRC cells. CRA also triggered humoral immunity, including tumor-binding activity and class-switching of antitumor autoantibodies, which contributed to its therapeutic efficacy in CRC mice. The mechanism of CRA-induced B cell activation was investigated by monitoring changes in cytokine profiles, tumor-infiltrating immune cells, reactive cells, and CRA-regulated molecules in the microenvironment. The active ingredients of CRA for B cell activation were also demonstrated to be two major compounds, 1,2-di-*O*-α-linolenoyl-3-*O*-(6-*O*-α-galactopyranosyl-β-galactopyranosyl)-*sn*-glycerol (designated CRDG) and glyceroglycolipid 1,2-di-*O*-α-linolenoyl-3-*O*-β-galactopyranosyl-*sn*-glycerol (dLGG, designated CRG in this study).

## Methods

### Preparation of the bioactive fraction CRA from *C. rabens* and purification of two major chemical constituents, CRDG and CRG, from CRA

The fresh or dried whole plant materials of *C. rabens* were extracted with alcohols, e.g., 50 to 99.5% ethanol or methanol to yield the total crude extract of *C. rabens*, which was further fractionated with reverse phase medium pressure liquid chromatography (RP-MPLC, Biotage, Uppsala, Sweden) to obtain a phytogalactolipid-enriched fraction, designated CRA. The mobile phase of MPLC was composed of primary alcohol (MeOH, EtOH, etc.) and distilled water. The CRA fraction was eluted by a high percentage of alcohols, e.g., 70 to 100%. The CRA fraction was subjected to high performance liquid chromatography system (HPLC, Agilent, Santa Clara, CA) with preparative column (reverse phase C18 column, 250 × 20 mm, Cosmosil, Kyoto, Japan) to collect pure CRDG and CRG. The mobile phase of HPLC was 98% MeOH with flow rate 5 mL/min to obtain CRDG and CRG at the retention time 24 min and 37 min, respectively. The purity and structural elucidation of CRDG and CRG were examined using analytical HPLC, high-resolution mass spectrometry and NMR spectroscopy (Hou et al., 2007).

### Cells

Mouse colorectal cancer CT26.CL25 cells were purchased from the Bioresource Collection and Research Center (BCRC, Hsinchu, Taiwan). CT26.CL25 cells were maintained in RPMI-1640 culture medium (Invitrogen, Carlsbad, CA) supplemented with 4.5 g/L glucose, 10 mM HEPES, 1.0 mM sodium pyruvate, 0.1 mM non-essential amino acids, 10% fetal bovine serum, and 1% penicillin–streptomycin (Invitrogen). Mouse metastatic castration-resistant prostate cancer MG-Cap A1 cells were kindly provided by Dr. Pei-Wen Hsiao of the Agricultural Biotechnology Research Center, Academia Sinica, Taipei, Taiwan, and maintained in DMEM supplemented with 10% fetal bovine serum, 10 μg/mL puromycin, 5 μg/mL blasticidin, and 1% penicillin–streptomycin.

### Animals

Female BALB/c mice aged 5 weeks were purchased from The National Laboratory Animal Center (Taipei, Taiwan). All animals were subjected to health monitoring and maintained on a 12:12-h light:dark cycle at a controlled temperature (22 ± 2 °C) and humidity (55 ± 10%) in a specific pathogen-free animal facility. Animals were acclimatized with free access to a rodent standard diet (LabDiet 5010, St. Louis, MO) and water. All procedures were approved by the Institutional Animal Care and Use Committee of Academia Sinica (approved protocol no. 20–12-1592) and followed the guidelines for the Use of Laboratory Animals (National Academy Press, Washington, DC).

### The therapeutic effects of CRA treatment in vivo

BALB/c mice were inoculated subcutaneously with 1 × 10^6^ CT26.CL25 cells in 100 μL of PBS. When the average tumor mass reached 100 mm^3^, the tumor-bearing mice were randomly divided into four groups. Animals were treated with 25, 50, and 100 mg/kg CRA via daily oral administration for 33 days. The tumor volumes were measured using a caliper every 3 days, and the volumes were calculated using the following formula: volume (mm^3^) = length × width × height. Before sacrifice, the sera of the mice were collected for experimental use.

### The anti-tumor effects of anti-sera in vivo

BALB/c mice were inoculated subcutaneously with 1 × 10^6^ CT26.CL25 cells in 100 μl PBS, and then treated with 100 μl anti-sera intravenously once a week. The tumor volumes were measured using a caliper every 3 days, and the volumes were calculated using the following formula: volume (mm^3^) = length × width × height. 

### In vivo cell depletion

B cells were depleted by monoclonal antibodies following the protocol of Dr.Yaron Carmi [14]. B cells were depleted by intraperitoneal (i.p.) injection of 300 μg anti-CD19 (1D3, BioXcell, West Lebanon, NH) and 300 μg anti-220 (RA3.3A1/6.1, BioXcell) monoclonal antibodies per mouse every 5 days for 3 weeks prior to tumor inoculation and thereafter. The extent of depletion of B cells was evaluated by IHC staining. Tumor tissue was randomly selected from depleted animals, embedded in paraffin and probed with rat monoclonal anti-B220 antibodies.

### Tumor-infiltrating lymphocyte isolation

Tumors were removed at the end of treatments. Freshly resected tumor tissues were mechanically ground in culture media and filtered through 40 μm nylon mesh. Then, tumor-infiltrating cells were isolated by density gradient centrifugation. In brief, cell pellets were resuspended in 80% Percoll (GE Healthcare, St Giles, UK), overlaid with 40% Percoll, and the interface was isolated after 30 min of centrifugation at room temperature.

### Phenotyping of splenic and tumor-infiltrating B cell subsets

The splenic and tumor-infiltrating lymphocytes of CRA-treated or untreated mice were harvested. B cell subsets, such as naïve (CD19^+^CD23^+^), activated (CD19^+^CD38^+^), memory B (CD19^+^CD27^+^) and plasma cells (CD19^− or low^ CD138^+^) were probed with specific monoclonal antibodies (BioLegend, San Diego, CA) and analyzed by flow cytometry (BD Biosciences). The indices of different types of B cell proportion were calculated as follows: (the number of splenocytes × the percentage of each B cell population in treated splenocytes)/(the number of splenocytes × the percentage of each B cell population in untreated splenocytes).

### Immunohistochemical staining

Tumors isolated from CRA-treated mice were fixed with 4% paraformaldehyde at 4 °C overnight. After washing with PBS, the tumor samples were placed in 30% sucrose at 4 °C overnight. Then, the tumor samples were embedded in OCT, and 10 μm sections were made and processed for immunohistochemical (IHC) staining. The sections were probed with Alexa Fluor 594-conjugated rat monoclonal anti-B220 (1:50 dilution; Biolegend) and PE-conjugated rat monoclonal anti-CD3 (1:50 dilution; Biolegend) antibodies. The sections were also probed with goat polyclonal anti-IL-21 (1:20 dilution; Thermo Fisher) at 4 °C overnight, and the detection antibodies were recognized using a Alexa Fluor 488-conjugated anti-goat IgG antibody (1:100 dilution; Thermo Fisher). The immune complexes in the sections were visualized and photographed at 200 × magnification using a Zeiss LSM 510 META confocal microscope (Carl Zeiss, Jena, Germany).

### Titers and Ig classes of antitumor autoantibodies

Blood was collected from CT26.CL25-bearing mice treated with PBS or 25, 50 or 100 mg/kg CRA, and the titers of CT26.CL25-specific antibodies in the sera were measured by the following method. The wells of 96-well culture plates were seeded with CT26.CL25 (10^6^ cells/well). On the following day, the cells were fixed with 4% paraformaldehyde, washed, and blocked with 300 µl of 2% skim milk in PBST (PBS buffer with 0.05% tween-20) for 1 h. One hundred microliters of 1000-fold diluted sera in PBS containing 0.5% skim milk were loaded into each well and incubated at room temperature for 2 h. After washing 3 times, 100 µl HRP-conjugated anti-mouse Ig antibody (1:5000 dilution; Sigma-Aldrich, St. Louis, MO) were added to each well and incubated for 1 h. After washing 3 times, 100 µL of NeA-Blue (Clinical Science Products, Mansfield, MA) was added to each well, incubated for 20 min and stopped using 100 µL 1 N HCl. The optical density was measured at 450 nm using an ELISA reader (Tecan, Mannedorf, Switzerland). The isotypes of specific anti-CT26.CL25 cell antibodies in anti-sera (1:1600 dilution) were determined by using HRP-conjugated specific anti-mouse IgM, IgG1, IgG2a, IgG2b, IgG3 or IgA antibodies (Acris, Herford, Germany).

### Immunoflourescence analysis of antisera binding to tumor antigens on the cell surface

CT26.CL25 or MG-Cap-A1 cells were incubated with 2 µL of normal sera, PBS antisera (control antisera) or CRA antisera for 1 h and detected with secondary antibodies conjugated to FITC (Molecular Probes, Eugene, USA). Antisera binding to tumor antigens on the surface of CT26.CL25 and MG-Cap-A1 cells were analyzed by flow cytometry (BD Biosciences).

### Co-immunoprecipitation assay

Five microliters of normal sera, control anti-sera or CRA anti-sera were mixed with 100 µl of protein A/G agarose (Merck Millipore, Darmstadt, Germany), and then covalently linked with disuccinimidyl suberate (DSS, Thermo Fisher Scientific). After washing, 5 µl of protein A/G agarose conjugated serum was incubated with 400 μL membrane proteins at room temperature. The immune complexes were then washed three times with lysis buffer and eluted with elution buffer (Thermo Fisher Scientific). The precipitated samples underwent protein identification in the Proteomics Core Lab (Institute of Plant and Microbial Biology, Academia Sinica) for analysis.

### Antigen identification by proteomics-based protein identification

The pull-down proteins were boiled at 95 °C for 10 min. The eluted proteins were loaded into an S-Trap spin column, and the trapped proteins were digested for 2 h at 47 °C by Lys-C and trypsin. The digested peptides were eluted from the column using 50 µl of 50 mM TEAB, 0.2% formic acid (FA), and 50% Acetonitrile (ACN). The solution was dried using a SpeedVac and the peptides were desalted using a SDBXC StageTip.

The dried peptides were re-dissolved in 10 μL of 0.1% FA for LC–MS/MS analysis. The Q-Exactive mass spectrometer (Thermo Fisher Scientific) coupled with an on-line nanoUHPLC (Dionex UltiMate 3000 Binary RSLCnano) was utilized for protein identification and analysis. An Acclaim PepMap 100 C18 trap column (75 µm × 2.0 cm, 3 μm, 100 Å, Thermo Scitific) and an Acclaim PepMap RSLC C18 nano LC column (75 µm × 25 cm, 2 µm, 100 Å) were used to deliver solvent and separate tryptic peptides with a linear gradient from 5 to 25% of ACN in 0.1% (v/v) FA for 60 min at flow rate of 300 nL/min. The raw files were searched against a Uniprot mouse database using Sequest and Mascot search engines in Proteome Discoverer (version 2.5).

### In vitro antibody-dependent cell cytotoxicity (ADCC), phagocytosis (ADCP), and complement-dependent cytotoxicity (CDC) assay

In in vitro ADCC assay, a total of 5 × 10^3^ CSFE-labeled CT26.CL25 target cells were pre-incubated with 2 μL of antisera. Mouse NK cells purified from mouse splenocytes were added at an effector with a target (E: T) cell ratio of 8:1. Assay plates were incubated at 37 °C for 4 h. Cytolysis was determined by an ELISA Reader (BioTek, Winooski, VT, USA).

For the ADCP assay, a total of 1 × 10^5^ labeled CSFE-labeled CT26.CL25 cells were incubated with 2 μL of antisera for 30 min and then added to 3 × 10^5^ macrophages. Four hours later, macrophages were probed with Alexa Fluor 647 conjugated rat monoclonal F4/80 antibodies (1:100 dilution; Biolegend). Phagocytosis was determined by counting double-labeled cells by flow cytometry (BD Biosciences).

For the CDC assay, a CT26.CL25 cell suspension containing 1 × 10^4^ cells in 100 μL of serum-free medium was mixed with 2 μL of antisera and incubated on ice for 30 min. Two hundred microliters of Gibco horse serum (Thermo Fisher) without heating was added and incubated at 37 °C for 2 h. Following washing with PBS, cell viability was determined by MTT assay.

### Cytokine assay

Mouse splenocytes isolated from CRA treated mice were seeded at 3 × 10^5^ cells/300 μL in each well of a 96-well microplate and then cultured for 72 h. Cell culture supernatants were collected and analyzed by a cytokine multiplex assay (Inflammation Core Facility, Institute of Biomedical Sciences, Academia Sinica, Taipei, Taiwan).

For analysis of IL-21 and IFN-γ concentration, splenocytes were seeded at 3 × 10^5^ cells in a 96-well culture plate and then treated with various doses of CRA or two compounds (CRG or CRDG) for 72. Splenic T cells were seeded at 3 × 10^5^ cells in a 96-well culture plate and then treated with various doses of CRA or two compounds (CRG or CRDG) for 24 h. The levels of IL-21 in the culture media were determined by the ELISA kit (R&D System, Minneapolis, MN) according to the manufacturer’s instructions.

### B cell proliferation and differentiation in vitro

Splenocytes (1 × 10^5^ cells/well) were labeled with CSFE for 37 °C for 15 min. Cells were then washed with PBS and suspended in fresh medium. CSFE labeled cells were treated with different concentrations of CRA for 72 h. B cell proliferation was analyzed by anti-CD19 antibody staining in CSFE-labeled cells. LPS (1 μg/mL) was used as a positive control. Another experiment of in vitro proliferation, splenocytes (1 × 10^5^ cells/well) were stimulated with 1 µg/mL of anti-CD40 antibody and 100 U/mL of IL-4, and treated with CRA for 72 h. B cell proliferation was determined as described above.

For B cell differentiation, after treating with CRA for 72 h, the proportions of plasmablasts (CD19^+^CD138^+^) and plasma cells (CD19^− or low^ CD138^+^) were probed with specific monoclonal antibodies (BioLegend) and analyzed by flow cytometry (BD Biosciences).

### Quantitative polymerase chain reaction

Splenocytes or splenic T cells were treated with CRA or components (CRG or CRDG), and then the mRNA expression of *Stat3, IL-21, Bcl6, cMaf* and *Aicda* was measured by quantitative polymerase chain reaction (qPCR). Briefly, total cellular RNA was extracted with Trizol reagent (Invitrogen) and reverse-transcribed into cDNA using the Superscript RT-kit (Invitrogen). Primers of *Stat3*, *Prdm-1*, *Bcl6*, *cMaf*, and *β-actin* were designed using Primer-BLAST on NCBI website. The primers for *Stat3* were: forward primer 5ʹ-AGGAGTCTAACAACGGCAGCCT-3ʹ and reverse primer 5ʹ-GTGGTACACCTCAGTCTCGAAG-3ʹ; the primers for *IL-21* were: forward primer 5ʹ-TAGACGCTCACGAATGCAGG-3ʹ and reverse primer 5ʹ-GTCTGTGCAGGGAACCACAA-3ʹ; the primers for *Bcl6* were: forward primer 5ʹ-CAGAGATGTGCCTCCATACTGC-3ʹ and reverse primer 5ʹ-CTCCTCAGAGAAACGGCAGTCA-3ʹ; the primers for *cMaf* were: forward primer 5ʹ-AGCAGTTGGTGACCATGTCG-3ʹ and reverse primer 5ʹ-TGGAGATCTCCTGCTTGAGG-3ʹ; The primers for *Aicda* were: forward primer 5ʹ-GCCACCTTCGCAACAAGTCT-3ʹ and reverse primer 5ʹ-CCGGGCACAGTCATAGCAC-3ʹ. All PCR reagents used to amplify the cDNA were purchased from Promega (Madison, WI, USA). Beta-actin was used as an endogenous control. The qPCR was performed using Fast SYBR Green Master Mix (Applied Biosystems, Waltham, MA, USA) and detected by ABI 7500 Fast Real-Time PCR System (Applied Biosystems). Relative fold expression was calculated by the 2^−ΔΔCT^ method using the following formulae: ΔCT (sample) = CT_target gene_ − CT_reference gene_.

### Western blot analysis

Splenocytes and splenic T cells were respectively treated with CRA for 72 h and 24 h and then lysed in RIPA lysis buffer. The protein concentration of the cell lysate was estimated with the Bradford protein assay using BSA as the standard. Total proteins (50 μg) were separated by SDS-PAGE using a 10% polyacrylamide gel and then transferred onto a nitrocellulose membrane. The membrane was blocked with 5% skim milk in phosphate buffered saline with Tween 20 (0.05% v/v Tween 20 in PBS, pH 7.2) for 1 h. The membranes were then incubated with primary antibodies such as anti-mouse STAT3 (1:1000, Cell Signaling), pSTAT3 (1:1000, Cell Signaling), anti-mouse Blimp-1 (1:1000, Cell Signaling) and anti-mouse AID (1:1000, Cell Signaling) at 4 °C overnight, followed by incubation with a horseradish peroxide-linked secondary antibody (1:10,000). The protein bands were visualized using the Amersham ECL Western Blotting Detection Reagents (GE Healthcare, Buckinghamshire, UK). The intensity of the chemiluminescence signal was quantified using the Biospectrum 815 Imaging System and Vision Works Software (UVP, Upland, CA).

### Drug docking

To investigate how CRG activates the STAT3/cMaf/BCL6 pathway, we predicted the target protein by the Super-PRED website (https://prediction.charite.de/index.php, accessed on March 7, 2023). CRG (red) was docked with IL-6 receptor α (IL-6Rα) by iGEMDOCK software (http://gemdock.life.nctu.edu.tw/ dock/igemdock.php). IL-6Rα (PBD code: 1N26) was selected from the Protein Data Bank (PDB). The parameters of iGEMDOCK were set as follows: population; 500, generation; 70, and solution; 100.

### Statistical analysis

The results were expressed as mean ± SD and analyzed using the SAS statistical software package (SAS Institute, Cary, NC). Kruskal–Wallis test was used for nonparametric data with a Dunn's multiple comparisons post hoc test of significance between individual groups. Differences with a *P* value of less than 0.05 were considered statistically significant.

## Results

### CRA inhibits tumor growth and activates systemic and tumor-infiltrating B cells

**Fig. 1:**
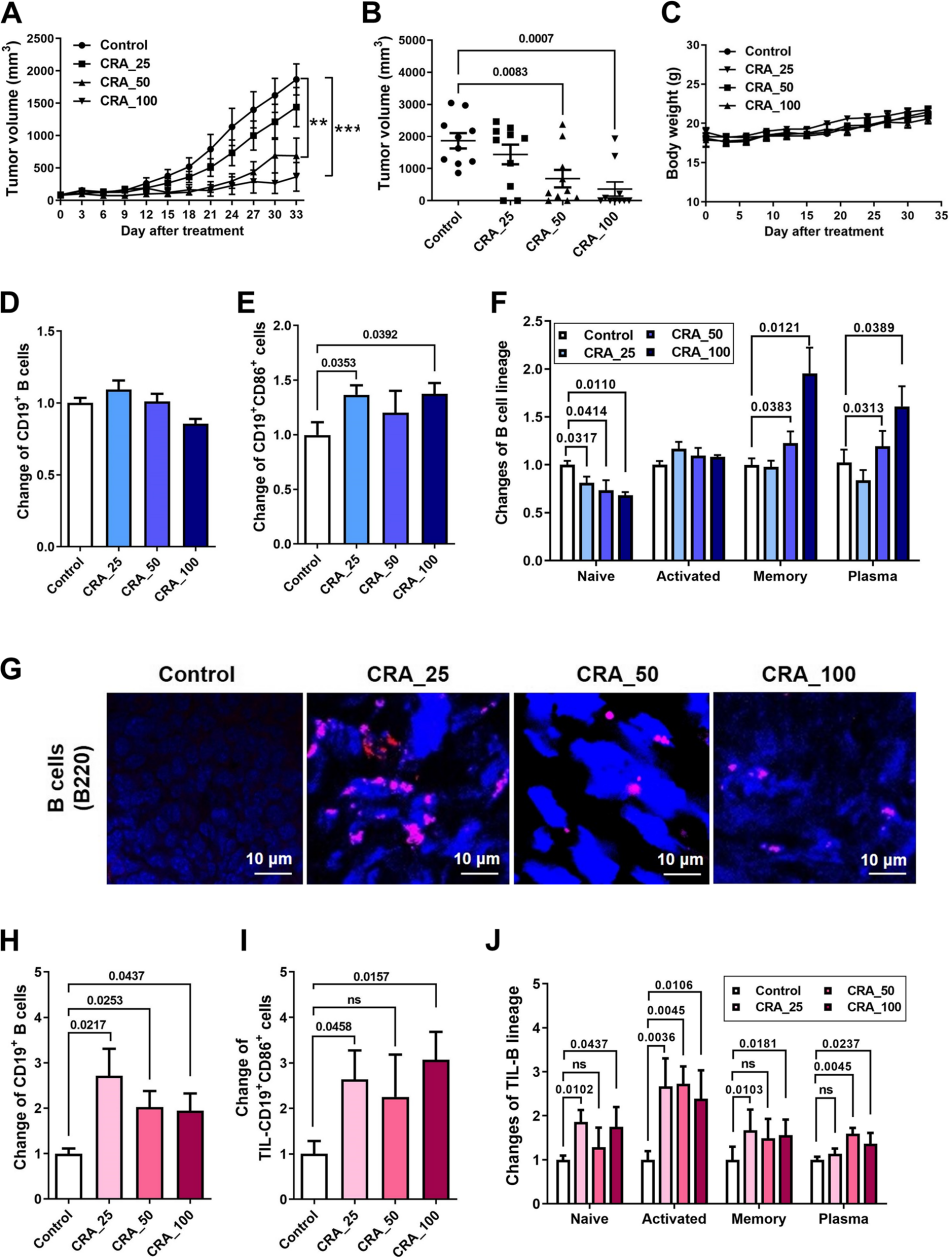
Effects of phytogalactolipid-enriched fraction CRA on tumor inhibition and B cell infiltration. Panel **A**. The inhibitory effects of CRA on tumor growth. Panel **B**. CRA inhibited tumor volumes in a dose-dependent manner at day 33. CT26.CL25 tumor-bearing mice were orally administrated with 25, 50, or 100 mg/kg of CRA every day. Tumor volumes were recorded every 3 days (*n* = 10). **: *P* < 0.01; ***: *P* < 0.001, compared to the control group. ns: no significant difference. Panel **C**. CRA did not affect the body weight in CRC tumor-bearing mice. After CRA treatment, Panel **D**. the proportion of splenic CD19^+^ B cells, Panel **E**. CD19^+^CD86^+^ B cells, and Panel **F**. different B cell subsets in splenocytes were determined (*n* = 10). Panel **G**. The expression of TIL-B cells (B220, red) in the tumor by IHC staining (magnification, 400 ×). Panel **H**. The proportion of TIL-CD19^+^ B cells, Panel** I.** TIL-CD19^+^CD86.^+^ B cells, and Panel** J.** Different TIL-B cell subsets were determined (*n* = 5)

To examine whether CRA triggered immunoregulatory activity for the suppression of tumor growth, the effects of oral injection with 25, 50 or 100 mg/kg of CRA into CT26.CL25 tumor-bearing BALB/c mice were monitored. After treatment for 21 days, 50 and 100 mg/kg of CRA significantly inhibited the growth of CT26.CL25 tumors in BALB/c mice (Fig. 1A). At the end of treatment (day 33), much smaller tumor masses were observed in the CRA_50 (40.9%) and CRA_100 (44.3%) groups than in the control (100%) group (Fig. 1B). Control and treatment mice exhibited similar body weights during the experiment (Fig. 1C).

To determine whether B cell activation is involved in the antitumor activity of CRA, the proportion of splenic CD19 positive B cells and their functional activity were analyzed. There was no significant difference between the control and CRA treatment mice in terms of the levels of splenic CD19 positive B cells (Fig. 1D), but CRA increased CD86 expression in splenic CD19 positive B cells (Fig. 1E). The different B cell subsets were further examined. CRA decreased the proportion of splenic naïve B cells, but increased the proportions of splenic activated, memory, and plasma cells (Fig. 1F). These results revealed that CRA did not change the levels of total CD19^+^ B cells but changed the proportion of B cell subsets. CRA increased the functional activity and promoted differentiation in systemic B cells. Using an immunohistochemistry (IHC) assay, it was shown that CRA treatments led to the infiltration of B cells into the tumor area (Fig. 1G). The activity and phenotype of TIL-B cells were also determined and characterized. Interestingly, CRA not only dramatically increased the infiltration of B cells but also increased CD86 expression on the surface of B cells, suggesting CRA effectively promoted activation of TIL-B (Fig. 1H and I). Furthermore, the numbers of TIL-activated B, memory B, and plasma cells also increased after CRA treatment, indicating CRA-induced B cell differentiation occurs in tumors (Fig. 1J). These results suggest that CRA-induced B cell activation and differentiation may play an adjuvant role in CRA-induced tumor inhibition.

### CRA increases the production and activity of anti-tumor autoantibodies

**Fig. 2:**
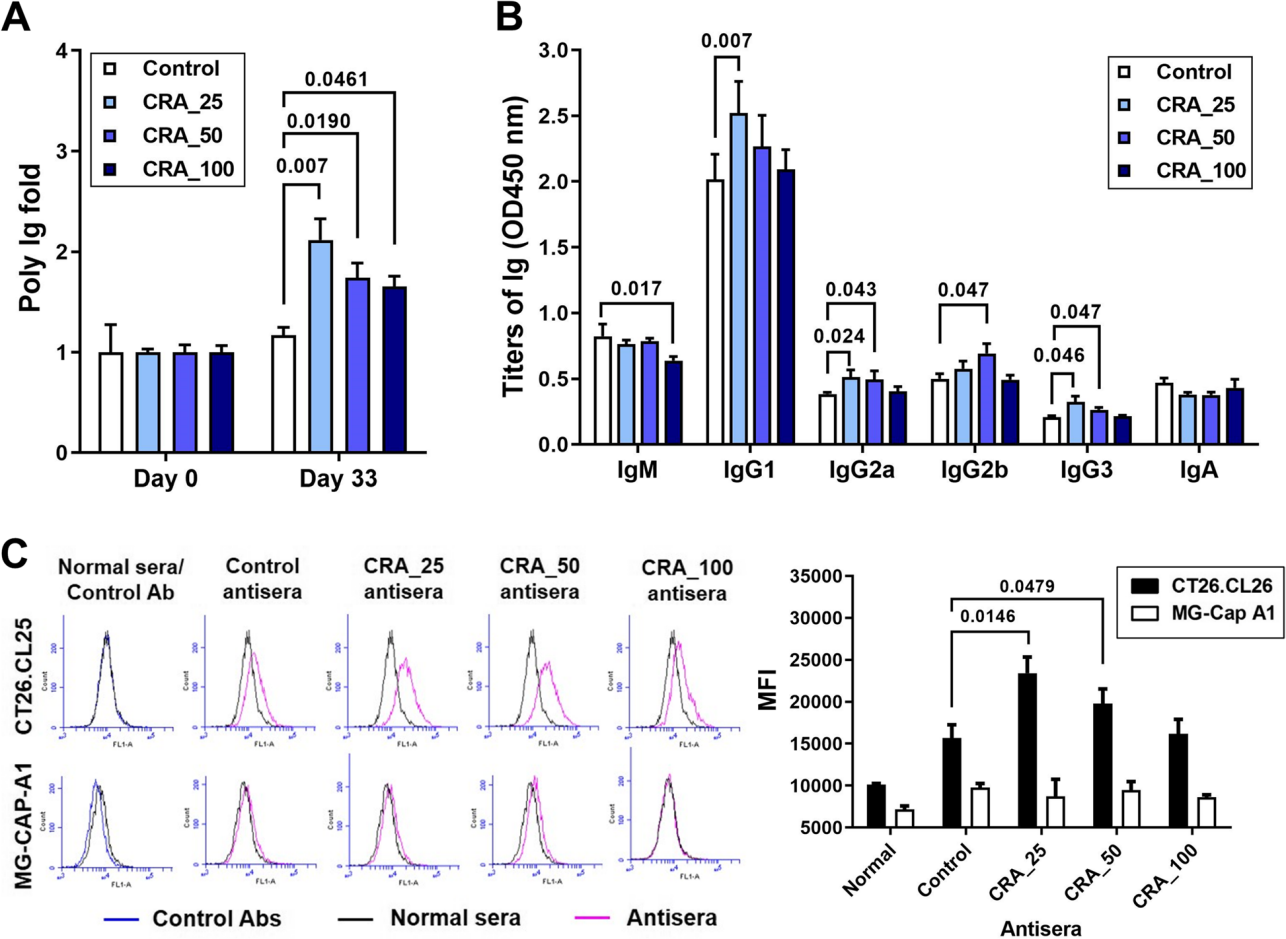
Titers and recognition of CRA-induced anti-tumor antibodies. Panel **A**. Titers of specific anti-CT26.CL25 cell antibodies for control or CRA antisera on days 0 and 33. Panel **B**. Effects of CRA on the class-switch of specific anti-CT26 cell antibodies. The titers of these antisera (1,000 × dilution) were measured for IgM, IgG1, IgG2a, IgG2b, IgG3, and IgA. Panel **C**. Tumor specificity of CRA antisera. Normal sera, control antisera, or CRA antisera bound to CT26.CL25 (black bar) or MG-CAP-A1 cells (white bar) were indirectly detected with FITC-conjugated goat anti-mouse IgG antibody

Characterization of the anti-sera revealed that the titers of specific anti-CT26.CL25 antibodies in CRA antisera were higher than in control antisera. Even a low dose (25 mg/kg) of CRA still increased the titers of anti-CT26.CL25 antibodies (Fig. 2A). The major classes of increased antibodies in CRA antisera were IgG1, IgG2a, IgG2b, and IgG3 (Fig. 2B). Like normal mouse sera, control antisera only somewhat recognized the CT26CL25 cells; however, the CRA antisera displayed a stronger binding affinity for the CT26CL25 cells (Fig. 2C). CRA antisera did not recognize the MG-Cap A1 cells (Fig. 2C) suggesting that CRA-induced antibodies in antisera have tumor specificity. Proteomics-based protein identification of the surface antigens bound by CRA-induced antibody is displayed in Table S1. CRA-induced antibodies can recognize multiple surface antigens on CT26.CL25 cells, such as heat shock protein, vimentin, ATP synthase, and glucose regulatory protein 78 (GRP78). These results demonstrate that CRA treatment enhances the production and class switching of tumor-specific antibodies that can recognize multiple antigens.

### CRA-activated humoral immune response suppresses tumor growth

**Fig. 3:**
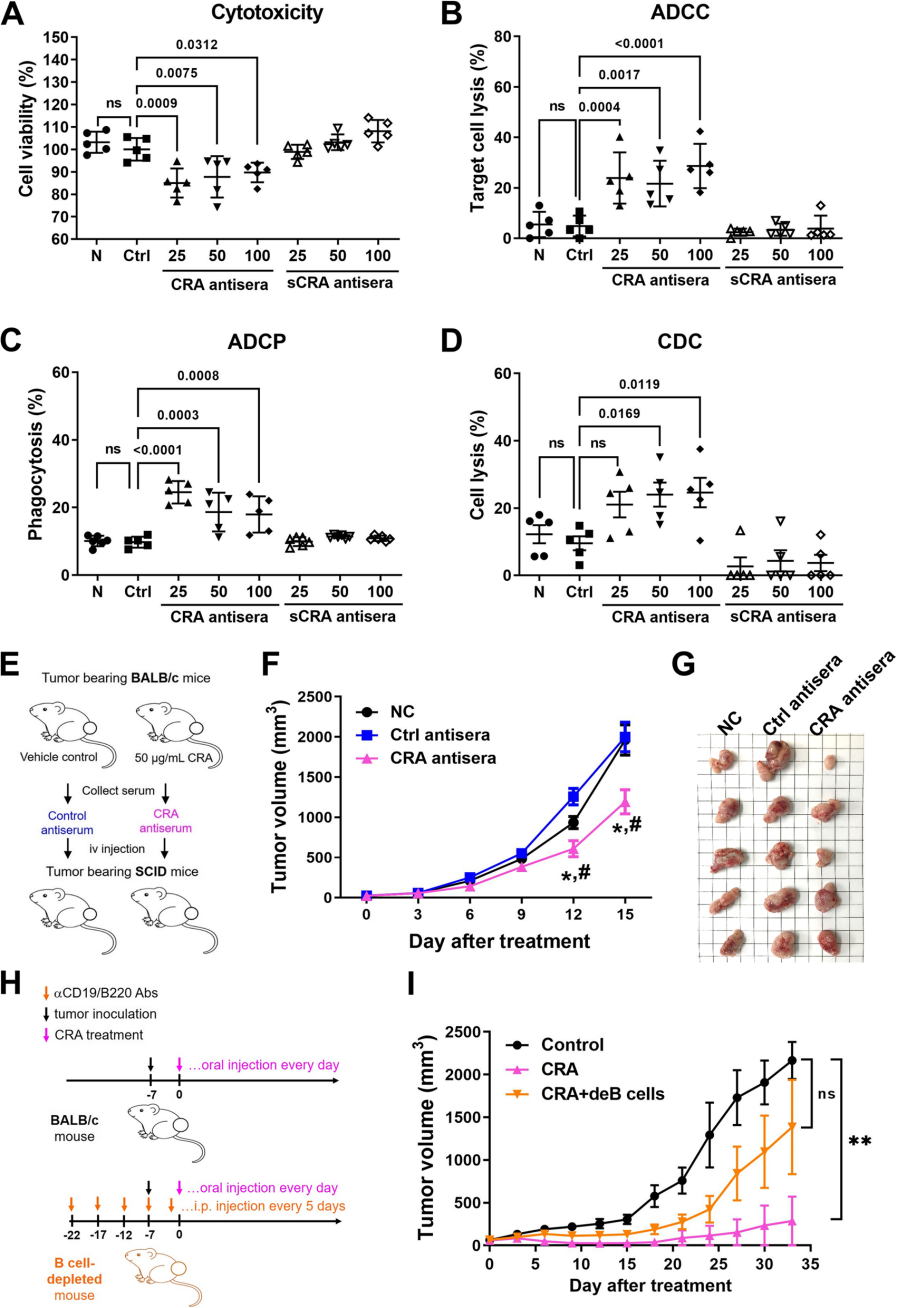
Antibody dependent cytotoxicity and antitumor activity of CRA-induced antisera collected from CT26.CL25 tumor-bearing mice. Panel **A**. Cytotoxicity of CRA antisera. CT26.CL25 cells were treated with 2 µL antisera and incubated for 48 h. Cell viability was determined by MTT assay. To prepare sCRA antisera, the anti-CT26.CL25 antibodies from CRA antisera were pre-subtracted by CT26.CL25 cell binding. The data are reported as the proliferation index. N: normal sera; Ctrl: control antisera; sCRA antisera: CRA antisera with the subtraction of anti-CT26.CL25 antibodies. Panel **B**. CRA antisera-mediated ADCC. CSFE labeled CT26.CL25 cells serving as target cells were incubated with mouse splenic NK cells (effector cells) in an E:T ratio of 8:1 with 2 µL normal sera or antisera for 4 h. Panel **C**. CRA antisera-mediated ADCP. RAW264.7 macrophages treated with unopsonized or opsonized CT26.CL25 cells (incubated with 2 µL antisera) for 4 h. The percentage of phagocytosis for normal sera and antisera is shown. Panel **D**. CRA antisera induced CDC activity. CDC activity of CRA antisera used horse complements. Cell lysis was determined after the addition of antisera for 4 h. Panel **E**. Schematic representation of animal study with CRA antisera. Panel **F**. Tumor volumes in the CT26.CL25 tumor-bearing SCID mice (*n* = 5/group) after treatment with 100 μL of control or CRA antisera weekly. *: *P* < 0.05, compared to the untreated group (NC); #: *P* < 0.05, compared to the control antisera group. Panel **G**. Image of tumors from control or CRA antisera treated mice. Panel **H**. Schematic representation of animal study with in vivo B cell depletion and CRA treatment. Panel **I**. Effects of CRA on tumor growth in immunocompetent or B cell-depleted BALB/c mice. B cell-depleted BALB/c mice (*n* = 5/group) bearing CT26 tumors were treated with 50 mg/kg of CRA by oral injection every week. Tumor volumes were measured every 3 days after treatment. **: *P* < 0.01, compared to the control group

To determine whether CRA-triggered humoral immunity is involved in the antitumor activity, cytotoxicity assay was performed on CRA antisera or control antisera. CRA antisera significantly inhibited cell proliferation in CT26.CL25 cells, whereas sCRA antisera (pre-subtract anti-CT26.CL25 cell antibodies) had no effect (Fig. 3C)A. CRA antisera also triggered ADCC, ADCP and CDC response to kill tumor cells (Fig. 3B, C and D). When the anti-CT26.CL25 cell antibodies were pre-subtracted, these three antibody-mediated cell death responses declined (Fig. 3B, C and D). Intravenous injection of CRA antisera into tumor-bearing SCID mice resulted in a significant reduction in tumor volume compared with the untreated group and control antiserum (Fig. 3E, F and G). These results demonstrated that the inhibition of tumor growth by CRA antisera is the result of CRA-induced antitumor antibodies in serum.

The superior tumor-suppressive effects of CRA resulted from the cytotoxicity of the drug and humoral immunity induction. When B cells were depleted, TIL-B also decreased and CRA-induced tumor suppression declined (Fig. 3H, I, and S2). These results also suggest that B cell-mediated humoral immune responses may play an adjuvant role in CRA-induced tumor suppression. Therefore, CRA-activated humoral immunity contributes to the therapeutic efficacy of CRA treatment in cancer therapy.

### Role of cytokine expression in CRA-induced B cell activation

**Fig. 4:**
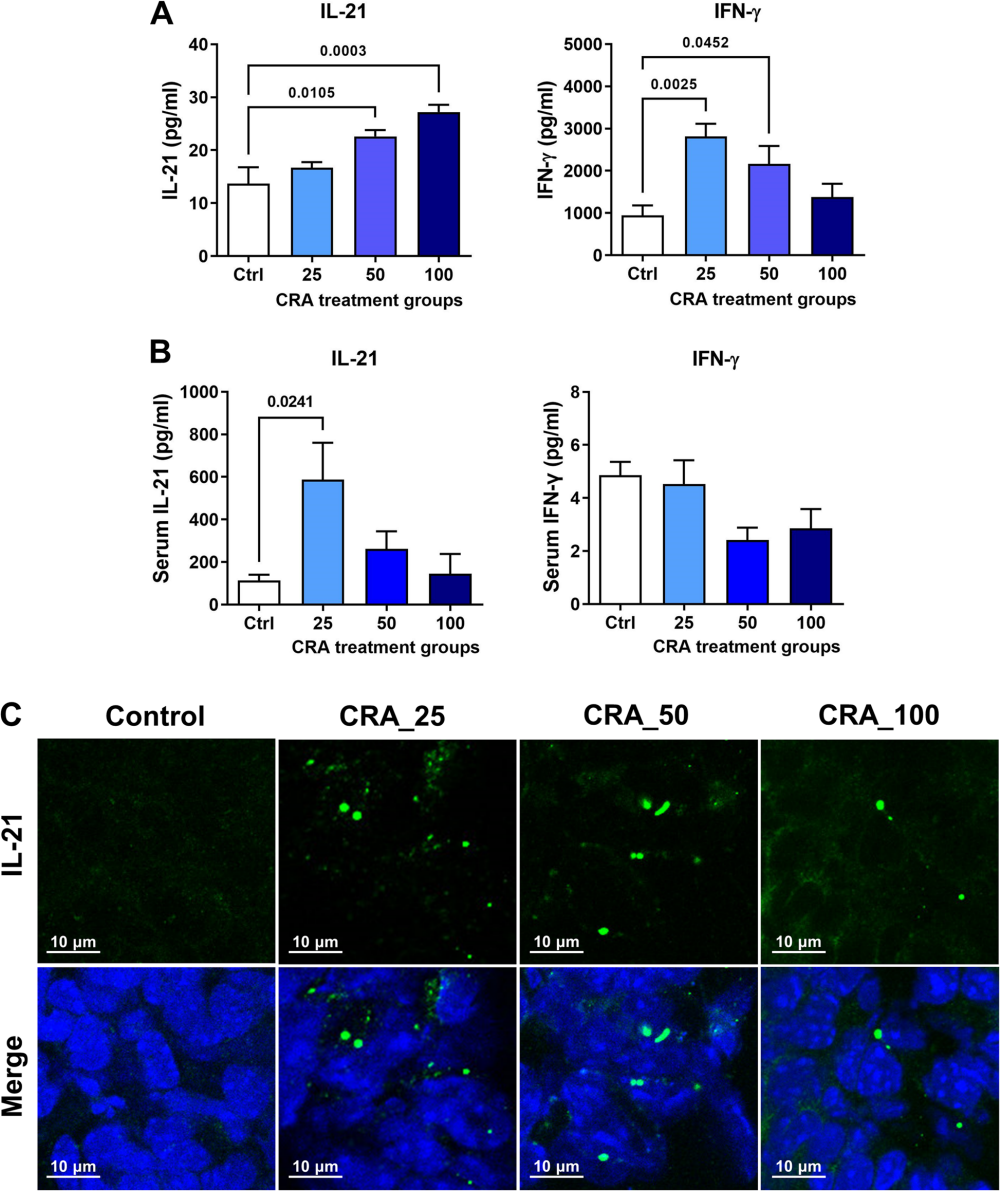
IL-21 and IFN-γ expression in splenocytes, sera, and tumors from the CRA-treated mice. Following different treatments, splenocytes harvested from CT26.CL25 tumor-bearing mice were cultured for 72 h. Then, Panel **A**. the release of IL-21 and IFN-γ in supernatants were analyzed. Panel **B**. Serum IL-21 and IFN-γ concentration. All values were expressed as the mean ± SD (*n* = 5). Panel **C**. The expression of IL-21 in the tumor from the CT26.CL25 mice after CRA treatment was observed by IHC staining (magnification, 400 ×)

Cytokine examination of splenocytes isolated from CRA-treated mice showed that levels of IFN-γ and IL-21 were significantly increased (Fig. 4A), but the levels of other cytokines did not change (Table S2). IL-21 levels in the sera of CRA-treated mice were also higher than that of the control group (Fig. 4B). In addition, it was found that CRA treatments enhanced the IL-21 expression in tumors (Fig. 4C). These results suggest that IL-21 may be an important cytokine in the modulation of B cell activation and differentiation.

### CRA promoted B cell differentiation in vitro

**Fig. 5:**
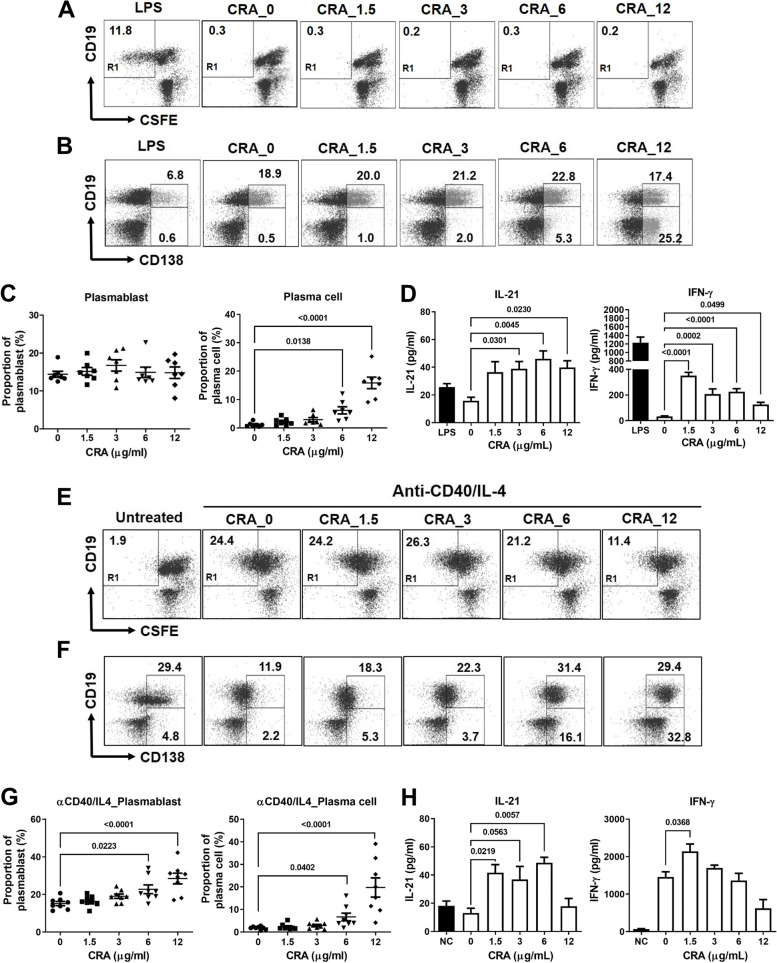
CRA induced B cell differentiation in splenocytes in vitro. Panel **A**. Cell proliferation of CD19^+^ B cells. LPS was used as a positive control for B cell proliferation. Panel **B**. Cell population of plasmablasts (CD19^+^CD138^+^) and plasma cells (CD19^− or low^ CD138^+^) in CRA-treated mouse splenocytes were analyzed by flow cytometry. Panel **C**. The proportion of plasmablasts (CD19^+^CD138^+^) and plasma cells (CD19^− or low^ CD138^+^). Mouse splenocytes were treated with serial dilution of CRA for 72 h. Panel **D**. CRA increased IL-21 and IFN-γ expression in splenocytes. Panel **E**. Cell proliferation of CD19^+^ B cells with anti-CD40 antibody (1 µg/mL), mouse IL-4 (100 U/mL) stimulation. Panel **F**. Cell population of plasmablasts (CD19^+^CD138^+^) and plasma cells (CD19^− or low^ CD138^+^) in CRA and/or anti-CD40/IL-4-treated mouse splenocytes were analyzed by flow cytometry. Panel **G**. The proportion of plasmablasts (CD19^+^CD138^+^) and plasma cells (CD19^− or low^ CD138^+^) in anti-CD40/IL-4 stimulated splenocytes. Panel **H**. CRA increased IL-21 and IFN-γ expression in anti-CD40/IL-4 stimulated splenocytes

To explore the effect of CRA on B cell activation, cell proliferation and differentiation following CRA treatment were measured in mouse splenocytes. CRA did not induce B cell proliferation in mouse splenocytes (Fig. 5A), but increased the proportion of plasma (CD19^low/−^CD138^+^) cells (Fig. 5B and C), indicating that CRA mainly promotes B cell differentiation rather than proliferation. CRA increased the IL-21 and IFN-γ release in splenocytes (Fig. 5D). In another set of in vitro studies, splenocytes were stimulated with anti-CD40 antibody and IL-4 and then treated with CRA. The results also showed CRA did not affect cell proliferation of splenic B cells (Fig. 5E) but accelerated B cell differentiation in anti-CD40 antibody and IL-4 stimulated splenocytes (Fig. 5F and G). In the culture media, the levels of IL-21 also increased in anti-CD40 antibody and IL-4 to stimulate splenocytes after CRA treatment (Fig. 5H).

**Fig. 6:**
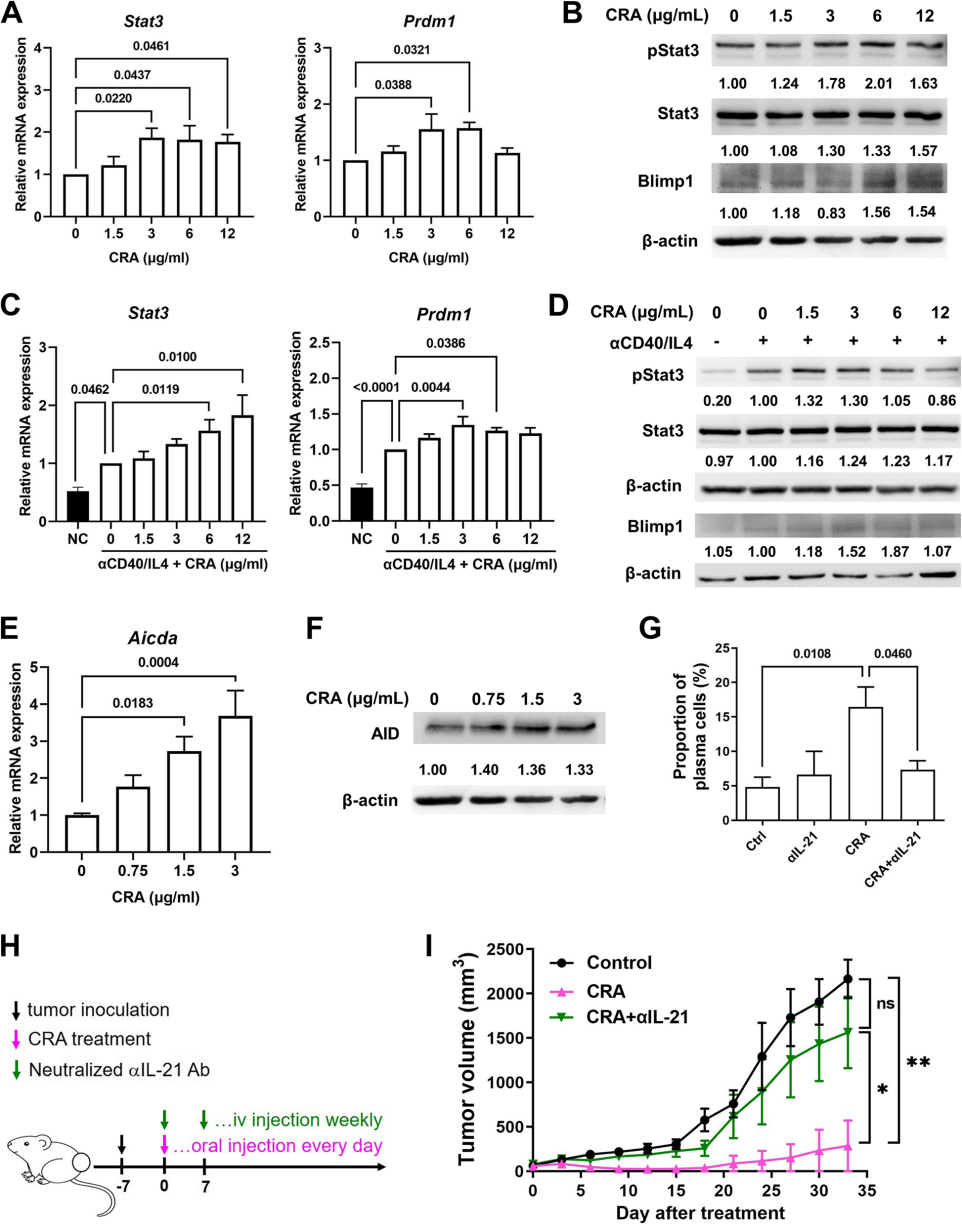
CRA induced B cell differentiation through upregulating the IL-21/IL-21R signaling pathway. Panel **A**. CRA increased the mRNA expression of *Stat3* and *Prdm1* in unstimulated B cells. Panel **B**. CRA increased the protein expression of pSTAT3, STAT3 and Blimp-1 in unstimulated B cells. Panel **C**. In anti-CD40/IL4-stimulated splenocytes, CRA also increased the *Stat3* and *Prdm1* mRNA expression and Panel **D**. STAT3, pSTAT3 and Blimp-1 expression in anti-CD40/IL4-stimulated splenocytes were enhanced after CRA treatment. Unstimulated or anti-CD40/IL4-stimulated splenocytes were treated with doses of CRA for 72 h, the expression of pSTAT3, STAT3 and Prdm1 was determined by qPCR and western blot. Panel **E**. CRA increased the mRNA expression of *Aicda.* Panel **F**. AID expression was upregulated in LPS-stimulated B cells after CRA treatment. Splenic B cells were treated with 10 μg/mL LPS and CRA for 120 h, the expression of AID was determined by qPCR and western blot. Panel **G**. Blockade of IL-21 by neutralizing anti-IL21 antibody inhibits CRA-induced B cell differentiation. Splenocytes were treated with CRA and 50 μg of anti-IL21 antibodies for 30 h. The proportion of plasma cells was analyzed by flow cytometry. Panel **H**. Schematic representation of animal studies of CRA treatment followed by serum IL-21 neutralization. Panel **I**. Blockade of serum IL-21 in CT26.CL25 tumor mice declined CRA-induced tumor suppressive effect. CT26.CL25 tumor-bearing mice were orally administrated with 50 mg/kg of CRA every day and i.v. injected with 800 ng of neutralizing anti-IL21 antibody weekly. Tumor volumes were determined every 3 days. *: *P* < 0.05; **: *P* < 0.01, compared to the CRA group (*n* = 5/group)

Subsequently, CRA treatment increased the gene and protein expression of STAT3 and Blimp which are downstream of the IL-21/IL-21 receptor signaling in B cells (Fig. 6A and B) or anti-CD40/IL-4 stimulated B cells (Fig. 6C and D). The expression of activation-induced cytidine deaminase (AID), which drives the processes of immunoglobulin somatic hypermutation (SHM) and class switch recombination (CSR), also increased after CRA treatment (Fig. 6E and F), suggesting that CRA promotes high-affinity antibody production. Therefore, CRA promotes B cell differentiation through the IL-21/IL-21R pathway and increases the affinity of anti-tumor antibodies.

To understand whether CRA can directly differentiate B cells or requires IL-21 mediation, splenic B cells were purified and then treated with CRA. In the absence of IL-21, CRA did not trigger the differentiation response of splenic B cells with or without anti-CD40 antibody and IL-4 stimulation (Fig. S2). Furthermore, CRA-induced differentiation declined when IL-21 released from CRA-treated splenocytes was neutralized by the neutralizing anti-IL-21 antibody (Fig. 6G). These results indicate that CRA-induced B cell activation requires the presence of IL-21, which can trigger IL-21/IL-21R signaling leading to B cell differentiation.

We further investigated whether IL-21 is a key regulator of CRA-induced humoral immunity against the CRC tumor in vivo, by evaluating tumor growth inhibition by CRA following IL-21 neutralization (Fig. 6H). When the levels of IL-21 in the blood diminished, the inhibitory effect of CRA on tumor growth in tumor-bearing mice declined (Fig. 6I and S3). Therefore, we concluded that IL-21 is a critical regulator of CRA-induced B cell differentiation, which mediates antitumor humoral immunity for CRC therapy.

### Role of T cells in CRA-induced B cell activation

**Fig. 7:**
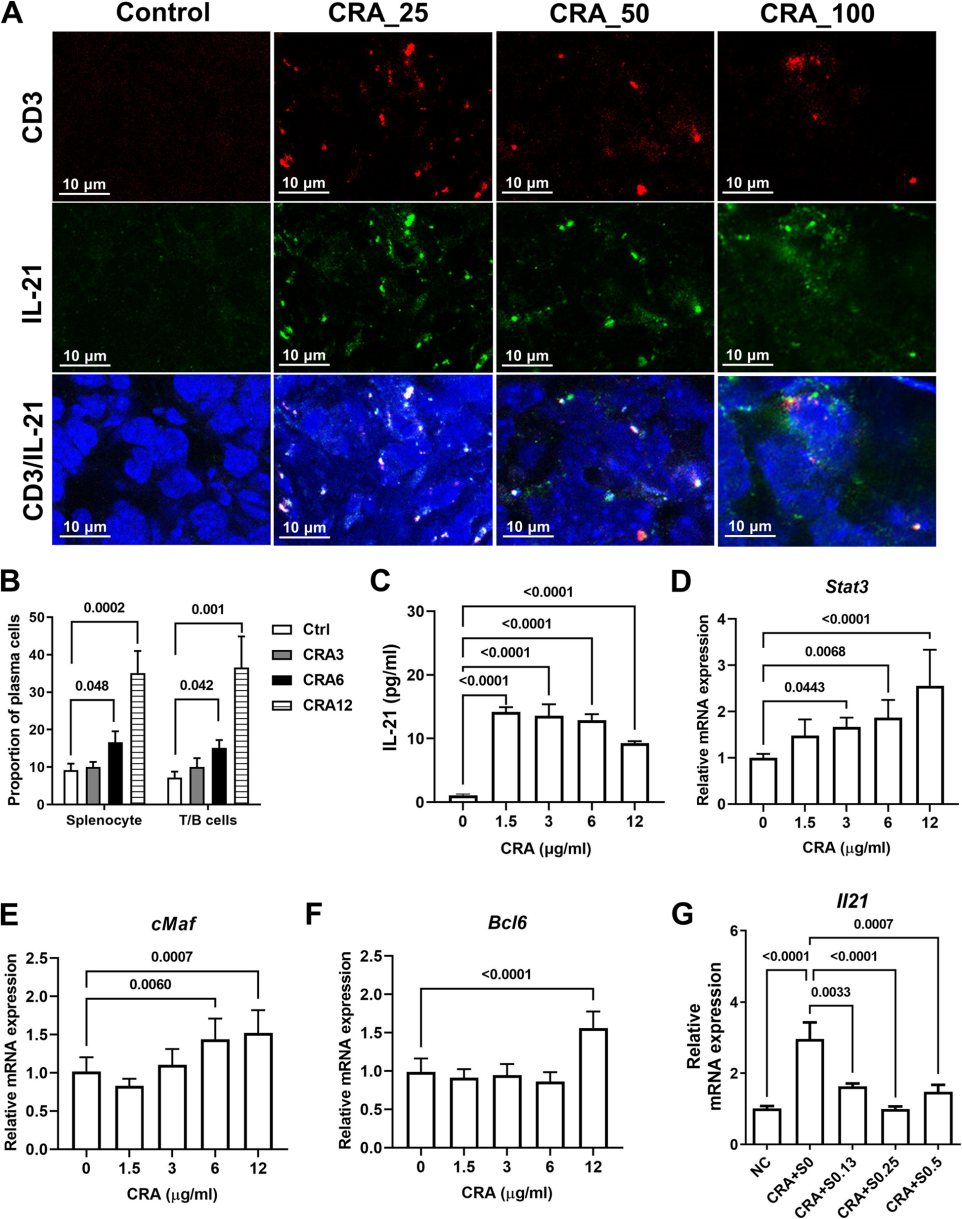
CRA promoted IL-21 release from T cells by upregulating STAT3/BCL6/cMaf pathway. Panel **A**. Tumor-infiltrating T cells expressed IL-21. CT26.CL25 tumor-bearing mice were orally administrated with 25, 50, or 100 mg/kg of CRA every day for 33 days. Co-localization between T cells (CD3, red) and IL-21 (green) in tumors were assessed by IHC (magnification, 400 ×). Panel **B**. CRA induced B cell differentiation in an in vitro co-cultural system of T and B cells. T and B cells at 1:2 ratio were co-cultured with 3 to 12 μg/mL of CRA for 72 h. The proportion of plasma cells was determined. Splenocytes were used as positive cell control. Panel **C**. CRA induced IL-21 release from T cells in vitro. After treating with CRA, the concentration of IL-21 in cell culture media of splenic T cells was determined by ELISA. The effects of CRA on Panel **D**. **E**. and **F**. *Stat3*, *cMaf*, and *Bcl6* expression in splenic T cells. Splenic T cells were treated with different doses of CRA for 24 h. The mRNA expression of *Stat3*, *cMaf*, and *Bcl6* was determined by qPCR. Panel **G**. Stattic suppressed CRA-induced IL-21 expression. Splenic T cells were pre-treated with different concentration (0.13 to 0.5 μg/mL) of Stattic for 1 h and then treated with CRA for 24 h. The mRNA expression of IL-21 was determined by qPCR

The immune cells that produce both IL-21 and IFN-γ should be the key reactive cells for CRA-induced B cell activation. It has been reported that T cells produce IL-21 through STAT3/cMaf/BCL6 signaling [15]. T cells were considered the major reactive cells targeted by CRA. The IHC assay showed that IL-21 can be locally produced from T cells in tumors (Fig. 7A). An in vitro co-culture experiment with T and B cells was used to prove that the T cells were the reactive cells targeted by CRA. When T cells were presented, the proportion of plasma cells increased in a dose-dependent manner (Fig. 7B). Splenic T cells were purified and then treated with CRA, and their ability to release IL-21 was determined. CRA not only increased IL-21 release (Fig. 7C) but also raised the expression of STAT3 and cMaf in a dose-dependent manner, whereas CRA increased the expression of BCL6 only at the highest dose (Fig. 7D, E and F). Thus, CRA induced T cells to produce IL-21 through the STAT3/cMaf/BCL6 pathway. The pathways involved in STAT3/cMaf/BCL6 after treatment with CRA were delineated through the use of a STAT3 inhibitor Stattic. When phosphorylation of STAT3 was downregulated by Stattic, the expression of IL-21 in CRA-treated T cells dramatically decreased (Fig. 7G). These results suggest that the STAT3/cMaf/BCL6 pathway mediates the IL-21 expression in CRA-treated T cells. Therefore, IL-21 released from CRA-activated T cells allows B cell differentiation and produces high titers and affinity of antitumor autoantibodies to inhibit tumor growth.

### Effects of active components of CRA on B cell differentiation

**Fig. 8:**
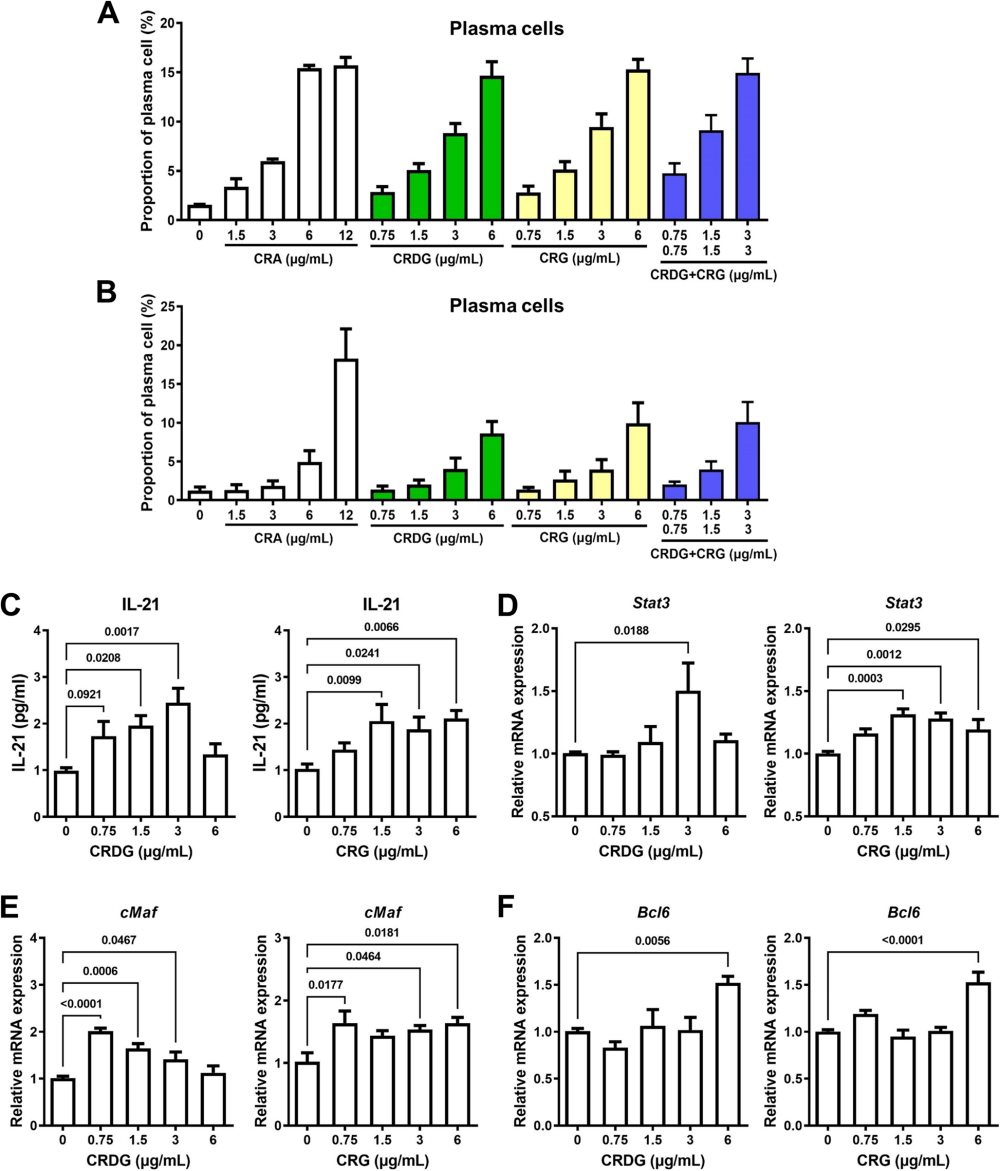
Two major compounds in CRA, CRDG and CRG, effectively induced B cell differentiation and triggered IL-21 production from T cells. Panel **A**. Cell population of plasma cells in CRA, CRDG, or CRG-treated mouse splenocytes. Panel **B**. Cell population of plasma cells in CRA, CRDG, or CRG-treated mouse splenocytes with anti-CD40 antibody/IL-4 stimulation. Panel **C**. Effects of CRDG or CRG on IL-21 release from splenic T cells. Panel **D**. **E**. **F**. The effects of CRDG or CRG on *Stat3*, *cMaf*, and *Bcl6* expression in splenic T cells. Splenic T cells were treated with different doses of CRDG or CRG for 24 h. The expression of *Stat3*, *cMaf*, and *Bcl6* was determined by qPCR

On the basis of HPLC data, CRA is composed of two major compounds, CRDG and CRG (Fig. S4). We have isolated and purified both compounds with more than 98% purity as judged by HPLC and NMR spectrometry, and investigated their effects on B cell differentiation by determining the proportion of plasma cells in splenocytes. Either CRDG or CRG all enhanced B cell differentiation (Fig. 8A and B). Furthermore, both CRDG and CRG induced splenic T cells to release IL-21 through the STAT3/cMaf/BCL6 pathway (Fig. 8C, D, E and F), suggesting that CRDG and CRG are the active compounds in good support of the CRA-promoted B cell differentiation.

### CRG activated STAT3/c-Maf/BCL6 pathway by interacting with IL-6R

**Fig. 9:**
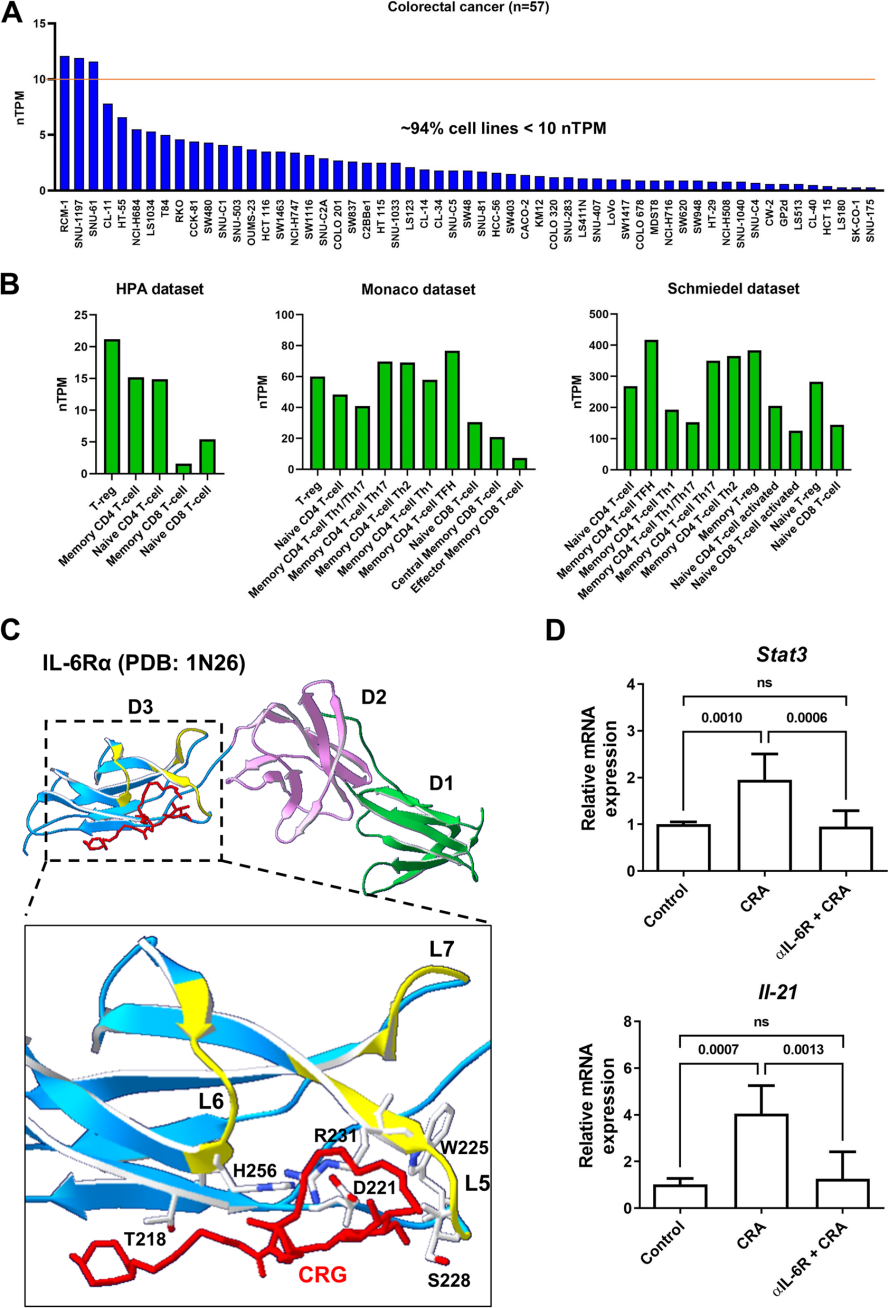
CRA activated STAT3 signaling by interacting with IL-6R. Panel **A**. IL-6R expression in CRC cancer cell lines. Gene expression of IL-6R in 57 CRC cancer cells obtained from the database of The Human Protein Atlas (https://www.proteinatlas.org/). Ninety-four CRC cell lines were shown their IL-6R expression below 10 nTPM. Panel **B**. IL-6R expression in T cells. Three datasets, including HPA, Monaco, and Schmiedel datasets showed high expression of IL-6R in different T cell subsets. Panel **C**. Docking of CRG into the D3 domain of IL-6Rα (PBD code: 1N26). The amino acids, including Thr218, Asp221, Trp225, Ser228, Arg231, and His256 that potentially interacted with CRG were highlighted. Panel **D**. Neutralizing IL-6R antibodies declined CRA-induced STAT3 activation and IL-21 production. Splenic T cells were pre-treated with 1 μg/mL anti-IL-6R antibodies for 30 min and then treated with 6 μg/mL CRA for 24 h. The STAT3 and IL-21 expression were analyzed by qPCR

To investigate how CRA activates the STAT3/cMaf/BCL6 pathway, we predicted the target protein by the Super-PRED website tool. Cytokine receptors are predicted as a target of CRA. In conventional, the binding of IL-6 to IL-6R and activation of the STAT3/c-Maf/BCL6 pathway is the main mechanism for IL-21 production in T cells. To make sure IL-6R is the possible target of CRG and CRDG, IL-6R expressed on CRC cells and T cells were analyzed from The Human Protein Altas. IL-6R expression on different T cell subsets was higher than on CRC cell lines (Fig. 9A and B). CRG was located in the space between loop 5 (L5) and loop 6 (L6) of D3 domain of IL-6R and interacted with Thr218, Asp221, Trp225, Ser228, Arg231, and His256 by hydrogen-bound (Fig. 9C). Ser228 and Arg231 on L5 are the key amino acids for IL-6 binding, indicating that CRA might bind to IL-6R and trigger IL-6R signaling to activate STAT3/cMaf/BCL6 pathway. To validate this mechanism, neutralizing anti-IL-6R antibodies were used to interrupt the binding between CRA and IL-6R. Neutralizing anti-IL-6R antibodies declined the CRA-induced STAT3 expression and phosphorylation and IL-21 production (Fig. 9D). These results suggest that binding to IL-6R is the main way that CRA activates the STAT3/c-Maf/BCL6 pathway.

## Discussion

**Fig. 10:**
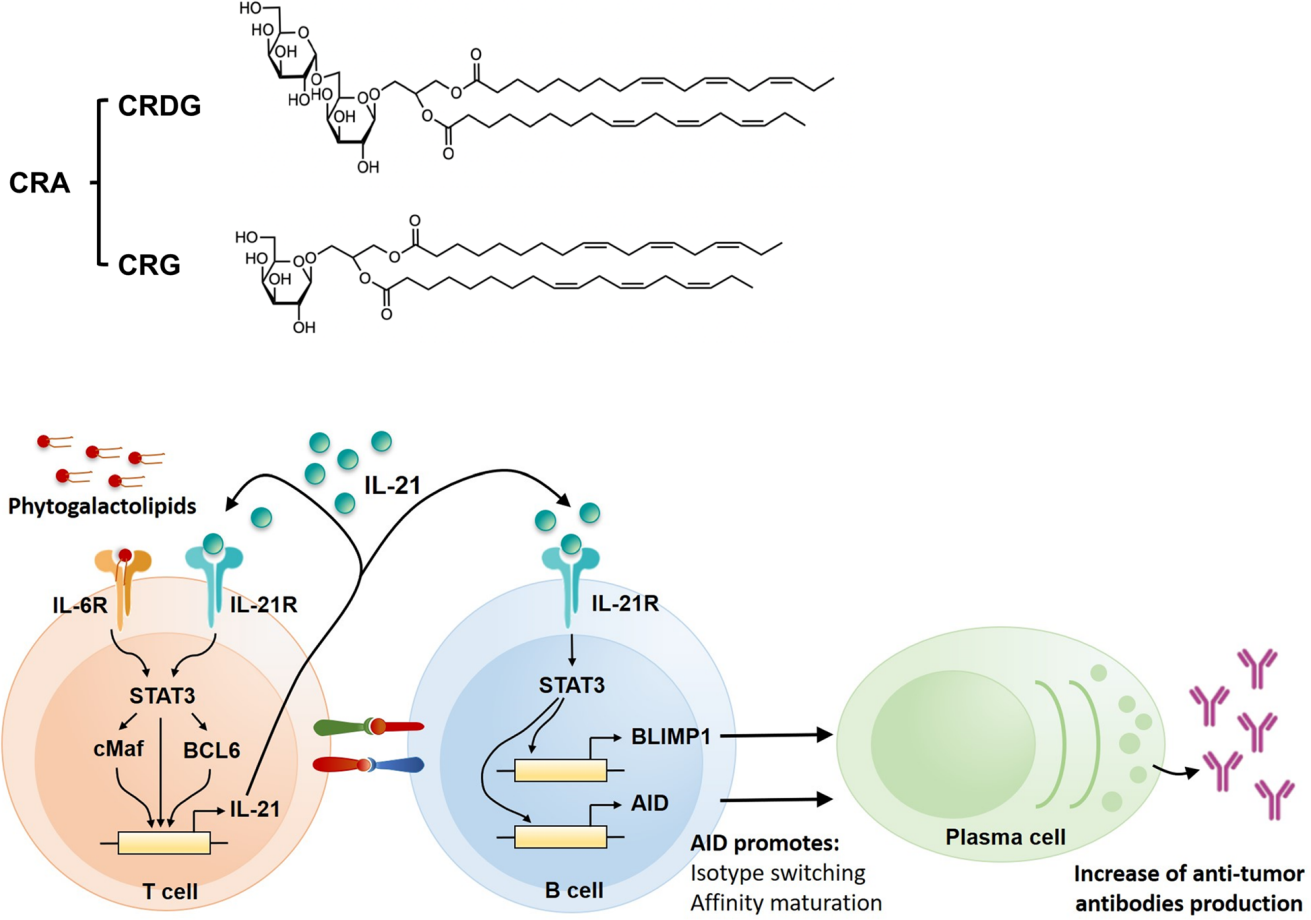
Schematic representation of the proposed mechanism of CRA-induced antitumor antibodies. CRA interacts with the IL-6R to trigger STAT3 pathways for IL-21 production in T cells. STAT3 signaling also leads to the upregulation of c-MAF and BCL-6 transcription factors, which then orchestrate a transcriptional program that upregulates genes encoding IL-21. CRA-induced IL-21 in T cells acts on naive B cells in conjunction with co-stimulatory signals to drive the differentiation of plasma cells subsequent to isotype switching and affinity maturation. IL-21 also upregulated BLIMP1 and then promoted the differentiation of plasma cells

This study revealed that a phytogalactolipid enriched fraction CRA from *C. rabens* (Benth.) S. Moore can abolish CRC tumor growth in BLAB/c mice through activating a humoral response to generate anti-tumor autoantibodies (AAs) with a higher titer, affinity, and cytotoxic activities (Figs. 1, 2 and 3). A proposed mechanism for CRA is shown in Fig. 10. CRA increased STAT3, cMaf, and BCL6 activity in T cells to induce the release of IL-21 (Fig. 7C-F), then it engaged the IL-21 receptors on the B cells (Fig. 6A-D) to activate STAT3/BLIMP-1 signaling to promote B cell maturation and differentiation. Blockade of CRA-induced IL-21, by neutralizing monoclonal anti-IL-21 antibody, significantly inhibited B cell differentiation (Fig. 6G). These results indicate that IL-21, triggered by CRA, is a critical cytokine for mediating B cell differentiation and antibody production. IL-21 is essential for B cell differentiation into plasma cells, promoting functional germinal centers and immunoglobulin production [16]. The predominant mechanism underlying IL-21–induced B-cell differentiation is STAT3-mediated induction of BLIMP-1, which is a transcriptional repressor for the generation of plasma cells and establishment of long-lived antibody response [17]. It has been reported that IL-21 can have both positive and negative effects on B cells in vitro. IL-21 increased the proliferation of murine splenic B cells that had been stimulated by anti-IgM and anti-CD40 [18]. Conversely, B cell proliferation induced by anti-IgM and IL-4 was inhibited by IL-21 [18]. In this study, CRA treatment did not affect proliferation in unstimulated splenocytes (Fig. 5A). However, in anti-CD40/IL4-stimulated splenocytes, B cell proliferation seems to be inhibited by CRA treatment (Fig. 5E). These results are similar to IL-21 addition to splenic B cells stimulated by anti-IgM and IL-4 in vitro [18], although the B cell differentiation was still promoted in both unstimulated and anti-CD40/IL4-stimulated cells (Fig. 5B, C, F and G). It is known that IL-21 signaling primes CD40-stimulated human naïve B cells to enhance their differentiation into plasmablasts via STAT3 [19]. Therefore, the role of CRA-induced IL-21 contributes to the promotion of B cell differentiation.

CRA enhanced titers of anti-tumor autoantibodies (Fig. 2A) and the increased antibody isotypes were IgG1, IgG2a, IgG2b, and IgG3 (Fig. 2B). Cytokines secreted by activated helper T cells determine Ig class-switching. CRA treatment-induced IL-21 and IFN-γ release (Fig. 4A and B). IL-21 modulates B cells to produce IgG1 and IgG3 antibodies whereas IFN-γ promotes IgG2a antibody production [20, 21]. SHM and CSR generate antibody diversity. Stimulation with anti-CD40 mAb and recombinant IL-21 induces activation-induced cytidine deaminase (AID) expression and causes CSR to produce IgG1 and IgG3 antibodies through induction of γ1 and γ3 germline transcripts and Sγ/Sμ switch circular DNA [18, 20]. IFN-γ produced by Th1 cells enhances the secretion of IgG2a. CRA-induced IL-21 and IFN-γ not only induce antibody class switching but also suggest that CRA activates both Th1 and Th2 responses.

IL-21 is produced by T cell populations, with the highest production by T follicular helper (Tfh) cells and Th17 cells, and lower levels produced by natural killer T (NKT) cells and CD8 + T cells [16]. In this study, CRA predominantly activated STAT3/c-Maf signaling and slightly activated Bcl6 signaling to produce IL-21. STAT3 expression in T cells was required for IL-21 production by multiple T helper subsets. These findings suggest that CRA is a STAT3 activator to increase c-Maf expression or directly activate c-Maf to induce IL-21 production. Furthermore, IL-21 also contributes to the growth and survival of T lymphocytes by both autocrine and paracrine of IL-21 [15]. Generally, IL-21 also affects the expression of transcription factors, such as STAT3, c-Maf and Bcl-6, which are central to Tfh development [15]. Interestingly, CRA activated the STAT3/cMaf/BCL6 pathway in T cells but had no effect (or even inhibition) in CRC cells (Fig. S5). Therefore, our data indicated that CRA triggers IL-21 release through activating STAT3, c-Maf, and Bcl6 signaling, in turn IL-21 promoted the development of Th subsets.

Cancer patients have been proven to generate autoantibodies in their sera that recognize their own tumor antigens. These autoantibodies do not provide therapeutic benefits for tumor elimination. In some cases, they accelerate tumor growth [22, 23]. Two types of anti-Trk autoantibody identified from the plasma of breast cancer patients show exactly opposite results. The agonist antibody has been shown to increase breast cancer cell growth, whereas the antagonist antibody inhibits growth [6]. Therefore, individual AAs may play a different role in cancer patients. In this study, CRA-induced AAs inhibited cell proliferation (Fig. 3A) and triggered antibody-dependent cytotoxicity (Fig. 3B-D). CRA-induced AAs recognize multiple antigens on the cell surface of CRC tumor cells, such as heat shock proteins, vimentin, ATP synthase, GRP78 and so on (Table S1). Cell surface vimentin has been suggested to define circulating tumor cells and be useful for cancer progression monitoring and relapse prediction [24–26]. Immunotherapies against cell surface vimentin have been developed to inhibit metastasis and prevent cancer recurrence [27, 28]. GRP78 manipulates cell proliferation, invasion, metastasis, and stemness in cancer [29]. GRP78 monoclonal antibody is a promising approach for killing cancer cells or modulating the tumor environment [30]. F1Fo-ATP synthase translocates outside the plasma membrane of tumor cells, although it was originally thought to be located only in the inner mitochondrial membrane. Cell surface F1Fo-ATP synthase performs extracellular ATP synthesis and regulates cell proliferation. Small molecule ATP synthase inhibitors are considered useful in cancer therapy. These reports demonstrated that when these surface antigens were blocked, tumorigenesis was effectively impeded. CRA-induced AAs not only recognized these antigens but also increase death in cancer cells. Therefore, CRA promotes the antitumor activity of AAs.

CRA improved the quality of autoantibodies by inducing a switch of the Ig class from IgM to IgG, thereby enhancing their binding affinity (Fig. 2B). In the body, IgG has a longer half-life in the blood. IgG efficiently increases the killing by NK cells and phagocytosis by macrophages. Thus, CRA increased the expression of AID (Fig. 6E and F), which induces SHM and CSR may be the reason that the CRA anti-sera causes more efficient inhibition of tumor growth than control anti-sera. CRA might enhance the process of somatic hypermutation in B cells that causes the autoantibodies in CRA anti-sera to have higher antigen binding affinities than those in control anti-sera, which results in the antitumor autoantibodies being able to stably bind on the surface antigens to provide cytotoxicity (Fig. 3A) or trigger more efficient ADCC and ADCP (Fig. 3B and C).

CRDG and CRG after oral treatment are likely to be cleaved through metabolism in the gastrointestinal system of animals. A previous study used human pancreatic lipolytic enzymes and duodenal contents to treat digalactosyldiacylglycerols (DGDG, relevant to CRDG) and observe that DGDG was converted into monogalactosyldiacylglycerols (MGDG, relevant to CRG), fatty acids and water-soluble galactose-containing compounds [31]. Similarly, for MGDG, free fatty acids were released after digestion. Our previous in vitro enzymatic assays data showed that galactose moiety of CRG could be cleaved by galactosidase, and linolenic acid moiety at either sn1 or sn2 position of the compound was cleavage by a lipase, suggesting a variety of metabolites of CRG might be formed in GI system of animals [11]. Thus, the bioactivity and pharmacological effects of CRDG and CRG in vivo are likely through the combinational effects of both compounds per se and their fatty acid metabolite.

## Conclusions

In summary, this study discovered a new pharmacological function of CRA on dominant humoral responses, involved T-cell-dependent induction of IL-21 through the IL-21R/STAT3/Blimp-1 pathway. CRA triggers a systemic and tumoral response to produce higher titer and better quality of antitumor autoantibodies than in the untreated mice resulting in elimination of tumors in animals.

## Supplementary Information

**Additional file 1: Table S1.** Tumor antigens recognized by CRA-antisera.

**Additional file 2: Table S2.** Effects of CRA on cytokines and chemokines expression in splenocytes isolated from CT26.CL25 tumor-bearing mice. **Fig. S1.** Effects of CRA on tumor growth in immunocompetent or B cell-depleted BALB/c mice. Panel A. Schematic representation of animal study with *in vivo* B cell depletion and CRA treatment. Immunocompetent or B cell-depleted BALB/c mice were inoculated subcutaneously with CT26.CL25 cells. When the average tumor mass reached ~80 mm^3^, the animals were treated with 50 mg/kg of CRA by oral injection every day. Panel B. B cell expression in tumor from CRA-treated immunocompetent or B cell-depleted mice. After treating with CRA, the expression of TIL-B cells (anti-B220, red) in the tumor was checked by IHC staining. Panel C. Anti-tumor effects of CRA in B cell-depleted BALB/c mice declined. *: *P* < 0.05; **: *P* < 0.01, compared to the control group. Mice with small tumors (<1000 mm^3^) or without tumors were counted, respectively. **Fig. S2.** Effects of CRA on B cell differentiation in splenic B cells *in vitro*. Panel A. CRA did not affect IL-21 and IFN-γ expression in splenic B cells. Panel B. Cell population of plasmablasts (CD19^+^CD138^+^) and plasma cells (CD19^- or low^ CD138^+^) in CRA-treated mouse splenic B cells were analyzed by flow cytometry. Panel C. The proportion of plasmablasts (CD19^+^CD138^+^) and plasma cells (CD19^- or low^ CD138^+^). Mouse splenic B cells were treated with serial dilution of CRA for 72h. ns: no significant difference, compared to the control group (*n*=6). M: cell culture medium. Panel D. CRA did not affect IL-21 and IFN-γ expression in anti-CD40/IL-4 stimulated splenic B cells. Panel E. Cell population of plasmablasts (CD19^+^CD138^+^) and plasma cells (CD19^- or low^ CD138^+^) in CRA and/or anti-CD40/IL-4-treated splenic B cells were analyzed by flow cytometry. Panel F. The proportion of plasmablasts (CD19^+^CD138^+^) and plasma cells (CD19^- or low^ CD138^+^) in anti-CD40/IL-4 stimulated splenic B cells. Splenic B cells were pre-stimulated with 1 µg/mL of anti-CD40 antibody and 100 U/mL of mouse IL-4. *: *P*<0.05; **: *P*<0.01, compared to the control group. NC: splenic B cells without anti-CD40/IL-4 and CRA treatment. **Fig. S3.** Effects of IL-21 neutralization on tumor growth in CRA-treated CT26.CL25 tumor mice. Panel A. Schematic representation of animal studies of CRA treatment followed by serum IL-21 neutralization. Panel B. Serum IL-21 concentrations in CRA-treated mice after IL-21 neutralization. Panel C. Blockade of serum IL-21 in CT26.CL25 tumor mice declined CRA-induced tumor suppressive effect. CT26.CL25 tumor-bearing mice were orally administrated with 50 mg/kg of CRA every day and iv injected with 800 ng of neutralizing anti-IL21 antibody weekly. Tumor volumes were determined every 3 days. *: *P* < 0.05, compared to the CRA group (*n*=5/group). Mice with small tumors (<1000 mm^3^) or without tumors were counted, respectively. **Fig. S4.** Chemical profile of CRA analyzed by reverse phase HPLC. Two major compounds CRDG (retention time = 10.2) and CRG (retention time = 12.9) are observed in CRA. A reverse phase C18 analytical column and diode array detector (DAD) set at 210 nm, 254 nm, and 360 nm were used. **Fig. S5.** Effects of CRA on STAT3/cMaf/BCL6 pathway in *colorectal cancer* cells. Panel A. Effects of low dose of CRA on gene expression of *STAT3*, *cMaf* and* BCL6* in mouse or human *CRC*cells. Mouse CT26.CL25 (while bars) and human HT-29 cells (gray bars) were treated with 1.5, 3, 6, or 12 μg/ml CRA (effective concentration for T cells) for 72 h. The gene expression of *STAT3*, *cMaf* and* BCL6 *was determined by qPCR. Panel B. The STAT3 expression and phosphorylation did not change after CRA treatment below 12 mg/ml. Panel C. Human HT-29 and COLO205 cells were treated high doses of CRA for 72 h. The protein expression of Stat3were determined by western blot.

## Data Availability

All data generated or analyzed during this study are included in this manuscript [and its supplementary information files].

## Abbreviations

STAT3: Signal transducer and activator of transcription 3
IL-21: Interleukin-21
Blimp-1: B-lymphocyte-induced maturation protein 1
TrkB: Tropomyosin receptor kinase B
GRP78: Glucose regulatory protein 78
IgG: Immunoglobulin G
ATP: Adenosine triphosphate
SCID: Severe combined immunodeficiency
IFN-γ: Interferon gamma
