# Supplementary material for: Phytogalactolipids activate humoral immunity against colorectal cancer
Source: J Exp Clin Cancer Res. 2023 Apr 21;42:95. doi: 10.1186/s13046-023-02660-x (PMC10120243; doi:10.1186/s13046-023-02660-x)
Supplement: Supplementary file 2 — Additional file 2: Table S2. Effects of CRA on cytokines and chemokines expression in splenocytes isolated from CT26.CL25 tumor-bearing mice. Fig. S1. Effects of CRA on tumor growth in immunocompetent or B cell-depleted BALB/c mice. Panel A. Schematic representation of animal study with in vivo B cell depletion and CRA treatment. Immunocompetent or B cell-depleted BALB/c mice were inoculated subcutaneously with CT26.CL25 cells. When the average tumor mass reached ~80 mm3, the animals were treated with 50 mg/kg of CRA by oral injection every day. Panel B. B cell expression in tumor from CRA-treated immunocompetent or B cell-depleted mice. After treating with CRA, the expression of TIL-B cells (anti-B220, red) in the tumor was checked by IHC staining. Panel C. Anti-tumor effects of CRA in B cell-depleted BALB/c mice declined. *: P < 0.05; **: P < 0.01, compared to the control group. Mice with small tumors (<1000 mm3) or without tumors were counted, respectively. Fig. S2. Effects of CRA on B cell differentiation in splenic B cells in vitro. Panel A. CRA did not affect IL-21 and IFN-γ expression in splenic B cells. Panel B. Cell population of plasmablasts (CD19+CD138+) and plasma cells (CD19- or low CD138+) in CRA-treated mouse splenic B cells were analyzed by flow cytometry. Panel C. The proportion of plasmablasts (CD19+CD138+) and plasma cells (CD19- or low CD138+). Mouse splenic B cells were treated with serial dilution of CRA for 72h. ns: no significant difference, compared to the control group (n=6). M: cell culture medium. Panel D. CRA did not affect IL-21 and IFN-γ expression in anti-CD40/IL-4 stimulated splenic B cells. Panel E. Cell population of plasmablasts (CD19+CD138+) and plasma cells (CD19- or low CD138+) in CRA and/or anti-CD40/IL-4-treated splenic B cells were analyzed by flow cytometry. Panel F. The proportion of plasmablasts (CD19+CD138+) and plasma cells (CD19- or low CD138+) in anti-CD40/IL-4 stimulated splenic B cells. Splenic B cells were pre-stim [file 13046_2023_2660_MOESM2_ESM.docx]

**Supplementary information**

**Table S2.** Effects of CRA on cytokines and chemokines expression in splenocytes isolated from CT26.CL25 tumor-bearing mice.

| Cytokines | Control | CRA  25 mg/kg | CRA  50 mg/kg | CRA  100 mg/kg |
| --- | --- | --- | --- | --- |
| IL-1α | ND | ND | ND | ND |
| IL-1β | 0.35 ± 0.13 | 0.79 ± 0.37 | 0.31 ± 0.27 | 0.32 ± 0.06 |
| IL-2 | 13.93 ± 8.02 | 15.87 ± 4.89 | 5.57 ± 2.74 | 10.43 ± 4.15 |
| IL-3 | 0.46 ± 0.40 | 1.07 ± 0.79 | 0.09 ± 0.13 | 0.36 ± 0.31 |
| IL-4 | 1.38 ± 0.30 | 1.13 ± 0.43 | 0.54 ± 0.18** | 0.45 ± 0.16** |
| IL-5 | 0.26 ± 0.33 | 0.24 ± 0.26 | 0.04 ± 0.08 | 0.36 ± 0.53 |
| IL-6 | 2.85 ± 0.51 | 3.84 ± 1.41 | 1.96 ± 0.61* | 2.16 ± 1.05 |
| IL-9 | ND | ND | ND | ND |
| IL-10 | 12.0 ± 4.5 | 11.8 ± 1.9 | 6.4 ± 3.6 | 9.4 ± 3.0 |
| IL-12 (p40) | 18.48 ± 9.69 | 21.82 ± 7.57 | 11.57 ± 3.53 | 15.84 ± 2.77 |
| IL-12 (p70) | 23.29 ± 5.85 | 25.04 ± 12.99 | 24.75 ± 15.30 | 32.44 ± 17.23 |
| IL-13 | ND | ND | ND | ND |
| IL-17A | 0.26 ± 0.07 | 0.40 ± 0.14 | 0.18 ± 0.17 | 0.33 ± 0.12 |
| **IL-21** | **13.70 ± 6.92** | **16.71 ± 2.32** | **22.60 ± 2.70*** | **27.21 ± 3.05**** |
| Eotaxin | ND | ND | ND | ND |
| G-CSF | 1.03 ± 1.39 | 1.94 ± 1.41 | 0.43 ± 0.70 | 0.70 ± 0.42 |
| GM-CSF | ND | ND | ND | ND |
| **IFN-γ** | **941.7 ± 532.8** | **2815± 673.2*** | **2064.5 ± 1123.7** | **1376.7 ± 706.0** |
| KC | ND | 0.56 ± 0.77 | 0.08 ± 0.18 | 0.90 ± 0.68 |
| MCP-1 | 32.14 ± 11.16 | 66.56 ± 32.51 | 17.26 ± 13.08 | 22.48 ± 12.98 |
| MIP-1α | 3.31 ± 1.26 | 2.22 ± 1.05 | 2.33 ± 0.37 | 2.74 ± 0.77 |
| MIP-1β | 17.90 ± 9.05 | 19.09 ± 6.61 | 10.23 ± 3.87 | 13.22 ± 6.29 |
| RANTES | 23.28 ± 11.93 | 20.27 ± 8.33 | 19.46 ± 3.80 | 19.54 ± 8.46 |
| TNF-α | 2.90 ± 0.46 | 2.90 ± 0.85 | 3.10 ± 1.44 | 3.10 ± 1.65 |

All values are mean ± SD (n=5). *: *P*<0.05; **: *P*<0.01 compared to control group.

ND: not detected.

IL-21 and IFN-γ were determined by ELISA while other cytokines were determined by a cytokine multiplex assay.

**
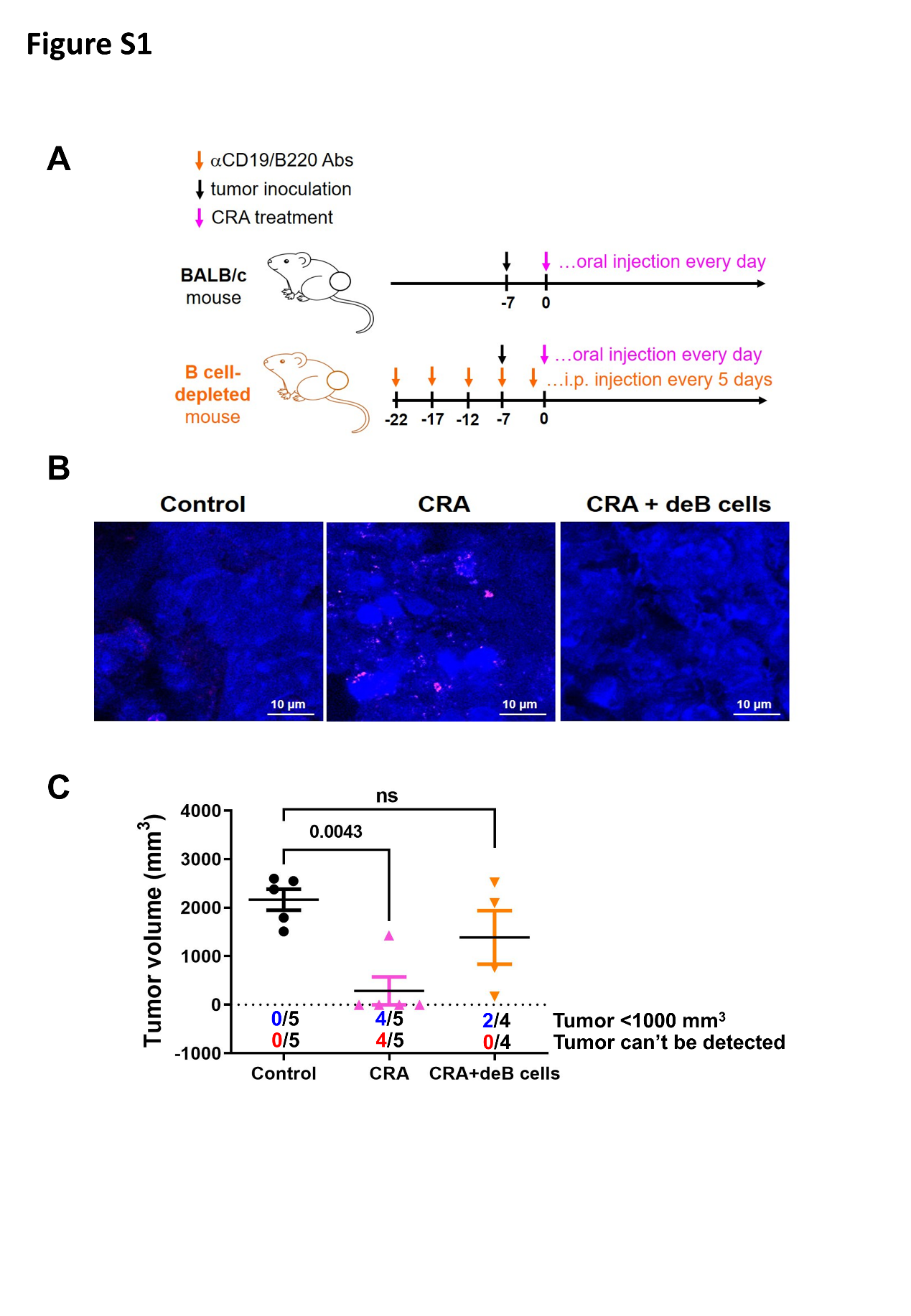
**

**Fig. S1** Effects of CRA on tumor growth in immunocompetent or B cell-depleted BALB/c mice. Panel **A.** Schematic representation of animal study with *in vivo* B cell depletion and CRA treatment. Immunocompetent or B cell-depleted BALB/c mice were inoculated subcutaneously with CT26.CL25 cells. When the average tumor mass reached ~80 mm^3^, the animals were treated with 50 mg/kg of CRA by oral injection every day. Panel **B.** B cell expression in tumor from CRA-treated immunocompetent or B cell-depleted mice. After treating with CRA, the expression of TIL-B cells (anti-B220, red) in the tumor was checked by IHC staining. Panel **C.** Anti-tumor effects of CRA in B cell-depleted BALB/c mice declined. *: *P* < 0.05; **: *P* < 0.01, compared to the control group. Mice with small tumors (<1000 mm^3^) or without tumors were counted, respectively.


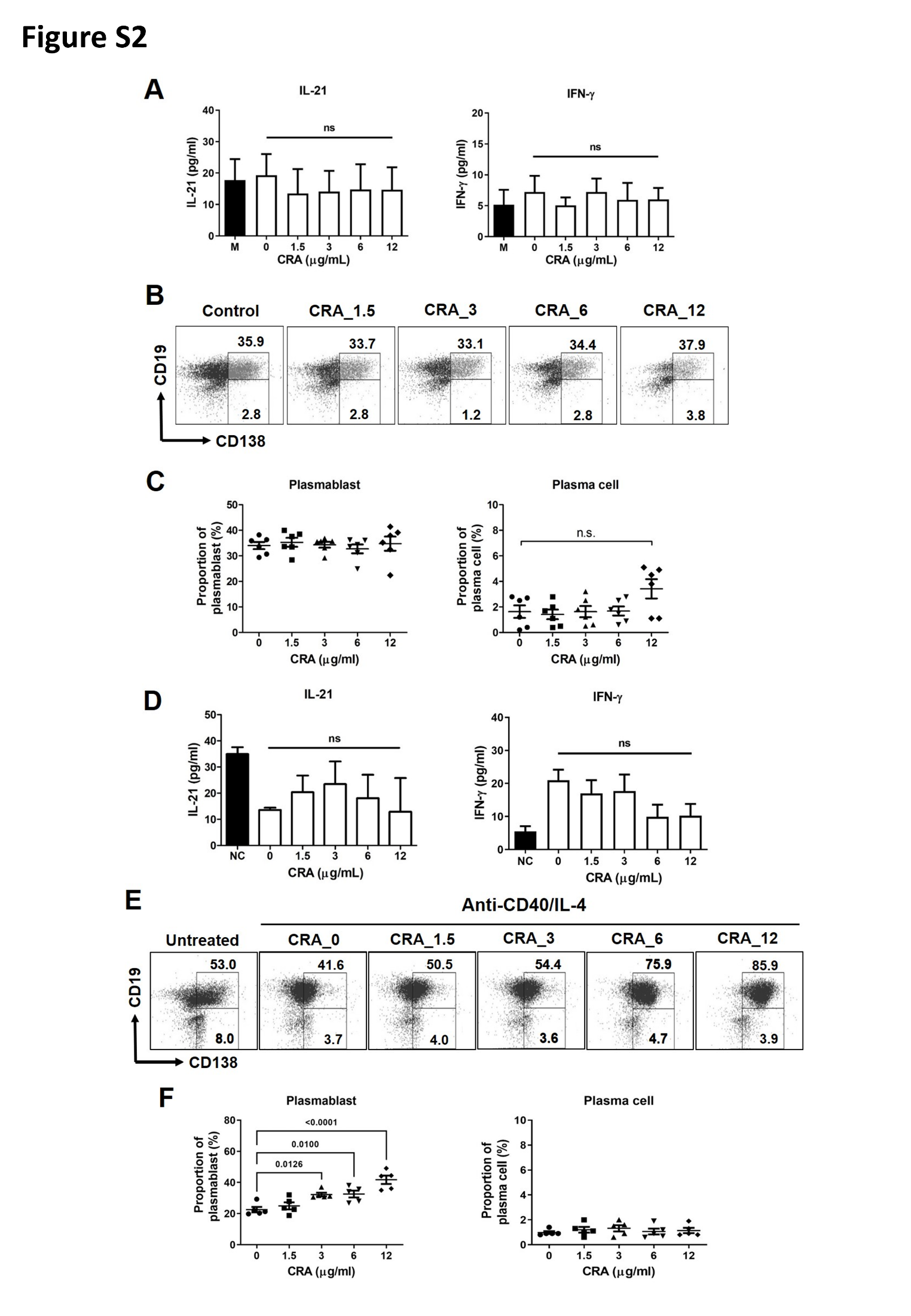


**Fig. S2** Effects of CRA on B cell differentiation in splenic B cells *in vitro*. Panel **A.** CRA did not affect IL-21 and IFN-γ expression in splenic B cells. Panel **B.** Cell population of plasmablasts (CD19^+^CD138^+^) and plasma cells (CD19^-^ ^or low^ CD138^+^) in CRA-treated mouse splenic B cells were analyzed by flow cytometry. Panel **C.** The proportion of plasmablasts (CD19^+^CD138^+^) and plasma cells (CD19^-^ ^or low^ CD138^+^). Mouse splenic B cells were treated with serial dilution of CRA for 72h. ns: no significant difference, compared to the control group (n=6). M: cell culture medium. Panel **D.** CRA did not affect IL-21 and IFN-γ expression in anti-CD40/IL-4 stimulated splenic B cells. Panel **E.** Cell population of plasmablasts (CD19^+^CD138^+^) and plasma cells (CD19^-^ ^or low^ CD138^+^) in CRA and/or anti-CD40/IL-4-treated splenic B cells were analyzed by flow cytometry. Panel **F.** The proportion of plasmablasts (CD19^+^CD138^+^) and plasma cells (CD19^-^ ^or low^ CD138^+^) in anti-CD40/IL-4 stimulated splenic B cells. Splenic B cells were pre-stimulated with 1 µg/mL of anti-CD40 antibody and 100 U/mL of mouse IL-4. *: *P*<0.05; **: *P*<0.01, compared to the control group. NC: splenic B cells without anti-CD40/IL-4 and CRA treatment.


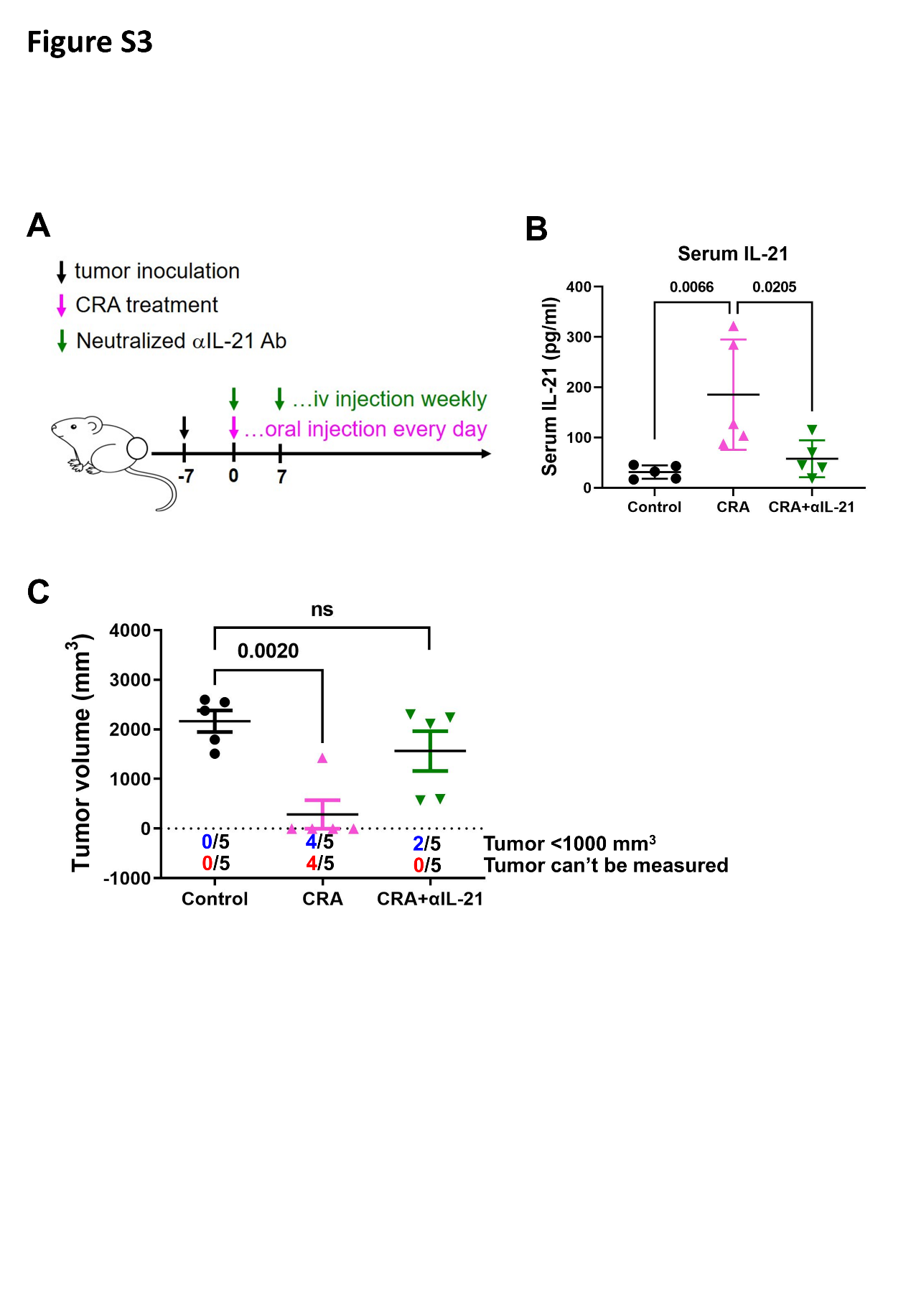


**Fig. S3** Effects of IL-21 neutralization on tumor growth in CRA-treated CT26.CL25 tumor mice. Panel **A.** Schematic representation of animal studies of CRA treatment followed by serum IL-21 neutralization. Panel **B.** Serum IL-21 concentrations in CRA-treated mice after IL-21 neutralization. Panel **C.** Blockade of serum IL-21 in CT26.CL25 tumor mice declined CRA-induced tumor suppressive effect. CT26.CL25 tumor-bearing mice were orally administrated with 50 mg/kg of CRA every day and iv injected with 800 ng of neutralizing anti-IL21 antibody weekly. Tumor volumes were determined every 3 days. *: *P* < 0.05, compared to the CRA group (n=5/group). Mice with small tumors (<1000 mm^3^) or without tumors were counted, respectively.


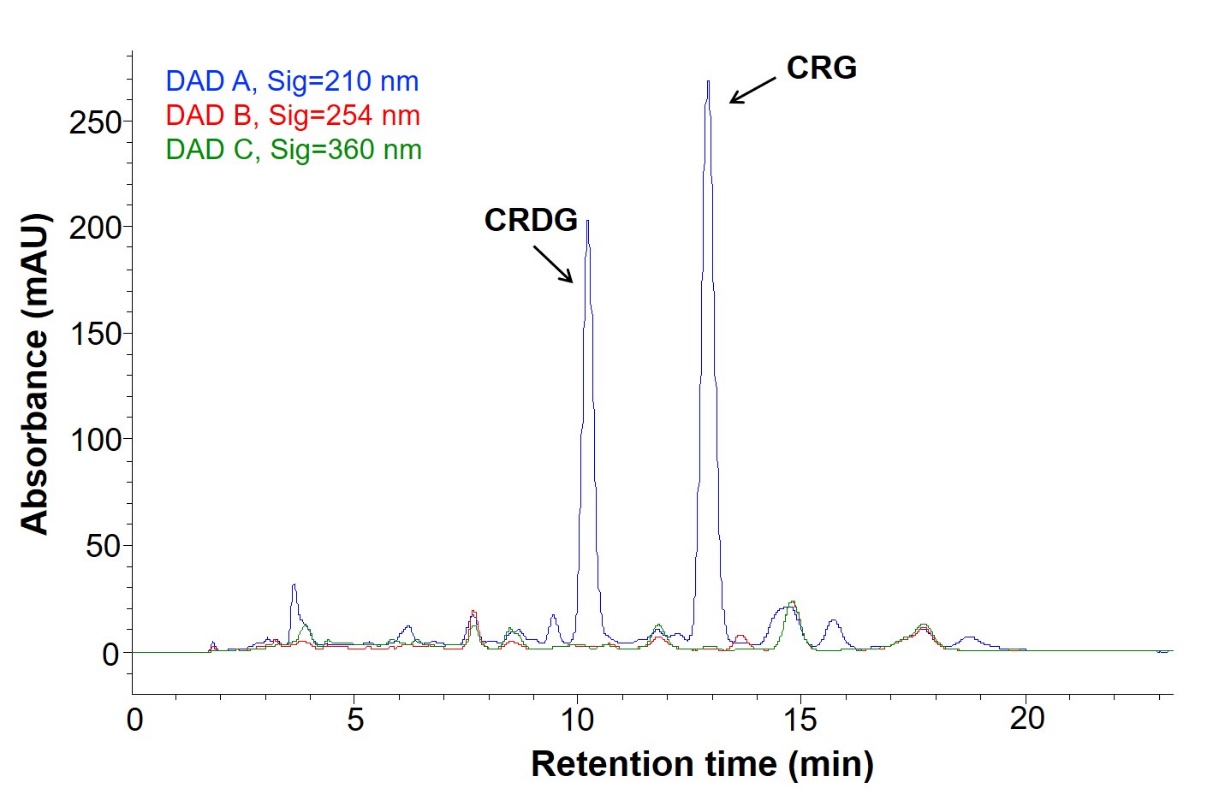


**Fig. S4** Chemical profile of CRA analyzed by reverse phase HPLC. Two major compounds CRDG (retention time = 10.2) and CRG (retention time = 12.9) are observed in CRA. A reverse phase C18 analytical column and diode array detector (DAD) set at 210 nm, 254 nm, and 360 nm were used.


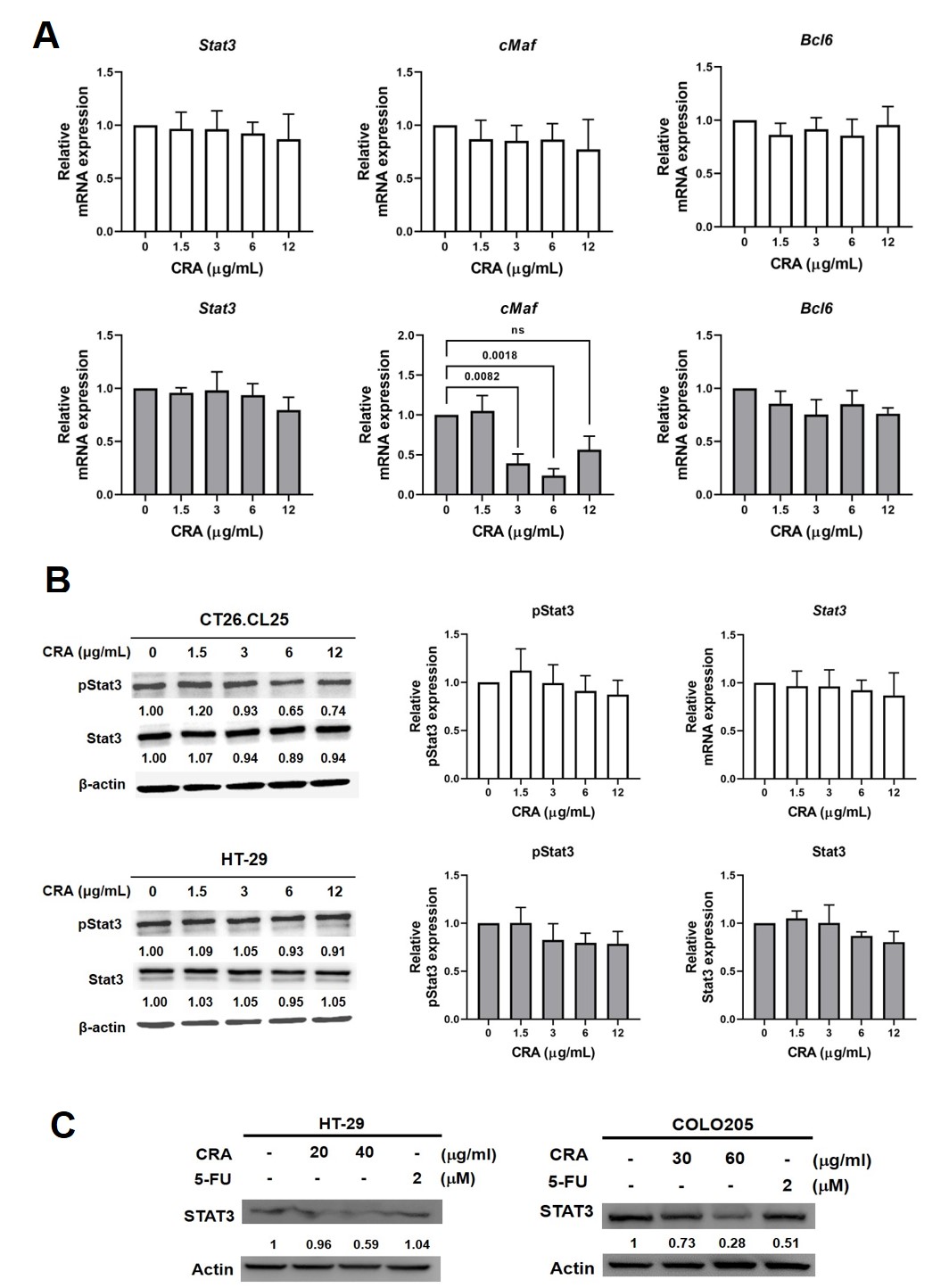


**Fig. S5** Effects of CRA on STAT3/cMaf/BCL6 pathway in colorectal cancer cells. Panel **A.** Effects of low dose of CRA on gene expression of *STAT3*, *cMaf* and *BCL6* in mouse or human CRC cells. Mouse CT26.CL25 (while bars) and human HT-29 cells (gray bars) were treated with 1.5, 3, 6, or 12 μg/ml CRA (effective concentration for T cells) for 72 h. The gene expression of *STAT3*, *cMaf* and *BCL6* was determined by qPCR. Panel **B.** The STAT3 expression and phosphorylation did not change after CRA treatment below 12 mg/ml. Panel **C.** Human HT-29 and COLO205 cells were treated high doses of CRA for 72 h. The protein expression of Stat3 were determined by western blot.
